# Protease resistance of infectious prions is suppressed by removal of a single atom in the cellular prion protein

**DOI:** 10.1371/journal.pone.0170503

**Published:** 2017-02-16

**Authors:** Henning Leske, Simone Hornemann, Uli Simon Herrmann, Caihong Zhu, Paolo Dametto, Bei Li, Florent Laferriere, Magdalini Polymenidou, Pawel Pelczar, Regina Rose Reimann, Petra Schwarz, Elisabeth Jane Rushing, Kurt Wüthrich, Adriano Aguzzi

**Affiliations:** 1 Institute of Neuropathology, University Hospital of Zurich, University of Zurich, Schmelzbergstrasse 12, Zurich, Switzerland; 2 Institute of Molecular Life Sciences, University of Zurich, Winterthurerstrasse 190, Zurich, Switzerland; 3 Institute of Molecular Biology and Biophysics, ETH Zurich, Otto-Stern-Weg 5, Zurich, Switzerland; 4 Department of Integrative Structural and Computational Biology and Skaggs Institute for Chemical Biology, The Scripps Research Institute, La Jolla, CA, United States of America; Van Andel Institute, UNITED STATES

## Abstract

Resistance to proteolytic digestion has long been considered a defining trait of prions in tissues of organisms suffering from transmissible spongiform encephalopathies. Detection of proteinase K-resistant prion protein (PrP^Sc^) still represents the diagnostic gold standard for prion diseases in humans, sheep and cattle. However, it has become increasingly apparent that the accumulation of PrP^Sc^ does not always accompany prion infections: high titers of prion infectivity can be reached also in the absence of protease resistant PrP^Sc^. Here, we describe a structural basis for the phenomenon of protease-sensitive prion infectivity. We studied the effect on proteinase K (PK) resistance of the amino acid substitution Y169F, which removes a single oxygen atom from the β2–α2 loop of the cellular prion protein (PrP^C^). When infected with RML or the 263K strain of prions, transgenic mice lacking wild-type (wt) PrP^C^ but expressing MoPrP^169F^ generated prion infectivity at levels comparable to wt mice. The newly generated MoPrP^169F^ prions were biologically indistinguishable from those recovered from prion-infected wt mice, and elicited similar pathologies *in vivo*. Surprisingly, MoPrP^169F^ prions showed greatly reduced PK resistance and density gradient analyses showed a significant reduction in high-density aggregates. Passage of MoPrP^169F^ prions into mice expressing wt MoPrP led to full recovery of protease resistance, indicating that no strain shift had taken place. We conclude that a subtle structural variation in the β2–α2 loop of PrP^C^ affects the sensitivity of PrP^Sc^ to protease but does not impact prion replication and infectivity. With these findings a specific structural feature of PrP^C^ can be linked to a physicochemical property of the corresponding PrP^Sc^.

## Introduction

Transmissible spongiform encephalopathies are fatal neurodegenerative diseases associated with the presence of prions [1]. It is well-established that prions contain aggregates of misfolded cellular prion protein (PrP^C^). Such aggregates have been termed “PrP^Sc^” because they were initially found in scrapie-affected sheep, or alternatively as “PrP^res^” to denote their extraordinary resistance to proteolytic digestion. Indeed, the carboxy-proximal core of PrP^Sc^ can largely withstand proteolysis with 500 μg/ml proteinase-K (PK) [2,3], and the detection of PK-resistant prion protein is commonly regarded as the definitive diagnostic method for prion diseases in humans and other species.

However, variably protease sensitive prionopathy (VPsPr) has been discovered in humans, characterized by atypical patterns of PrP^Sc^ detected by Western blots [4]. PK-treated PrP from VPsPr shows bands in the range from 8 to 17 kDa, which are not observed in sporadic Creutzfeldt-Jakob Disease (sCJD). Furthermore, recent studies have identified prion diseases with high titers of prion infectivity in the absence of protease resistance [5]. These observations point to heterogeneity in the structural arrangement of PrP aggregates, with looser aggregates being more solvent-accessible and therefore prone to proteolysis.

In all mammals, PrP^C^ displays a long flexible tail (FT, residues 23–125; numbering for mouse PrP) and a globular domain (GD, residues 126–231) whose C-terminal end is membrane-tethered through a glycosyl phosphatidyl inositol anchor. The GD carries also two glycosyl moieties [6,7]. A wealth of knowledge is available on the structure of wild-type (wt) [8–19] and many mutant forms of PrP^C^ [14–17,20–22]. Although the general architecture of the GD is conserved, there are substantial differences in the loop linking the β2 strand with the α2 helix (β2–α2 loop). The residue Y169 within this loop is strictly conserved, and its replacement with glycine or alanine results in a major change of the loop structure [20,21].

Here we investigate the phenotypes arising in transgenic mice that express a minimal modification of the residue in position 169, i.e., replacing tyrosine (Y) by phenylalanine (F). It should be stressed, in view of the unexpected results obtained in these experiments, that this variation of the protein structure includes the elimination of a single oxygen atom from the wt structure of mouse PrP.

## Results

### Prion infection of *Tg*(MoPrP^169F^) and wild-type C57BL/6 mice results in similar phenotypes

**Fig 1:**
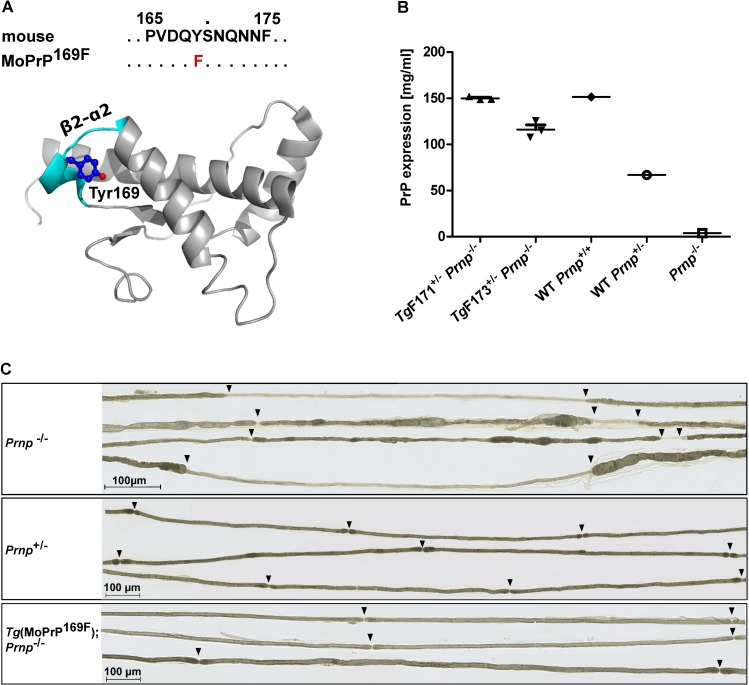
Cellular MoPrP^169F^ is biologically equivalent to wild-type MoPrP^C^. (A) Sequence comparison of the β2–α2 loop region in wild-type (wt) mouse PrP and *Tg*(MoPrP^169F^), and ribbon presentation of mPrP(121–231) at 37°C (PDB ID 2L39). The amino acid sequence is depicted by one-letter symbols, conserved residues by dots, and the amino acid substitution is indicated in red. In the 3D structure the backbone is shown in grey, except that the β2–α2 loop is highlighted in cyan. Tyr169 is blue, with the hydroxyl group in red. (B) Quantification, using a FRET assay, of PrP expression levels in wt *Prnp*^*+/+*^, *Prnp*^*+/-*^, *Prnp*^*-/-*^ and two lines of *Tg*(MoPrP^169F^) mice, denoted as *Tg*F171 and *Tg*F173. FRET analysis was performed using the anti-PrP antibodies POM2, labeled with europium, and POM1, labeled with APC. Each symbol represents a biological replicate. Data are represented as the mean ± SEM. (C) Sciatic nerve myelination in 19-month old *Prnp*^-/-^, *Prnp*^+/-^, and *Tg*(MoPrP^169F^);*Prnp*^-/-^ littermates. Teased fiber preparations from *Prnp*^-/-^ mice showed segmental demyelination with shortened internodes and marked thinning of the myelin sheaths. There were no myelination abnormalities in *Prnp*^+/-^ and *Tg*(MoPrP^169F^);*Prnp*^-/-^ mice. Scale bar indicates 100 μm. Arrowheads indicate nodes of Ranvier.

To investigate the biological effect of the hydroxyl group at position Y169 (Fig 1A), we generated mice expressing MoPrP^169F^ using a "half-genomic" *Prnp* minigene construct [23]. B6D2 hybrid zygotes were microinjected with the hg-MoPrP^169F^ construct, and four independent transgenic mouse lines were generated. Lines 171 and 173 were selected for mating with PrP-deficient *Prnp*^-/-^ mice [24] over multiple generations, resulting in mice expressing only the transgene (MoPrP^169F^;*Prnp*^-/-^) or both the transgene and endogenous PrP^C^ in a hemizygous state (MoPrP^169F^;*Prnp*^+/-^).

Line 171 showed the highest transgene expression in the brain, with mice hemizygous for the transgene showing a PrP protein expression slightly higher than C57BL/6 wild-type (wt) mice (Fig 1B, S1 Fig and S12 Fig), and was therefore chosen for further experiments. Clinical and histological examinations failed to reveal any pathological phenotype or deviation from the characteristics of wt mice up to the age of 2 years and 6 months (S2 Fig). In particular, in 19 month old littermates, myelination of the sciatic nerve was similar to that of hemizygous *Prnp*^+/-^ mice, whereas *Prnp*^-/-^ mice displayed a chronic demyelinating neuropathy (Fig 1C) [25]. Since the latter is a universal phenotype common to all investigated *Prnp*^-/-^ mouse strains, we concluded that MoPrP^169F^ was physiologically equivalent to wt PrP^C^.

**Fig 2:**
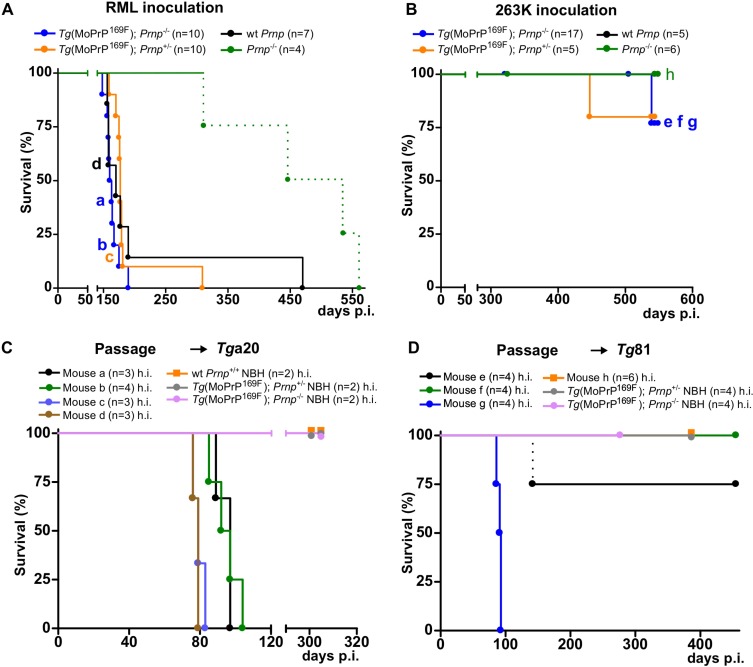
Prion infection of *Tg*(MoPrP^169F^) and wild-type mice. (A) Survival of RML-inoculated *Tg*(MoPrP^169F^);*Prnp*^*-/-*^, *Tg*(MoPrP^169F^);*Prnp*^*+/-*^, and wild-type control mice with median survival times of 161.5, 177 and 170 days, respectively. The letters a, b, c and d within the graph indicate individual mice that were used for further passaging. *Prnp*^-/-^ mice were used as control. Dashed line: deaths by intercurrent disease. (B) Survival of *Tg*(MoPrP^169F^);*Prnp*^*+/-*^, *Tg*(MoPrP^169F^);*Prnp*^*-/-*^, wild-type and *Prnp*^*-/-*^ mice after intracerebral (i.c.) inoculation of brain homogenates from 263K-inoculated *Tg*81 mice. Mice did not develop any clinical signs of a prion disease within 500 days post inoculation. Letters e, f, g and h within the graph indicate mice that were used for subsequent passaging. (C) Passage of brain homogenates from RML-infected *Tg*(MoPrP^169F^);*Prnp*^*-/-*^ (mice a and b), *Tg*(MoPrP^169F^);*Prnp*^*+/-*^ (mouse c) and wt *Prnp*^+/+^ (mouse d) animals into *Tg*a20 mice evoked a prion disease with median survival times between 79 and 97 days. *Tg*a20 mice re-inoculated with noninfectious brain homogenates (NBH) from either *Tg*(MoPrP^169F^);*Prnp*^*-/-*^, *Tg*(MoPrP^169F^);*Prnp*^*+/-*^ or *Prnp*^*-/-*^ mice did not show any signs of a prion disease (h.i. = heat inactivated). (D) Same as (C), but passage of brain homogenate from 263K-infected *Tg*(MoPrP^169F^);*Prnp*^*-/-*^ (mice e, f and g) animals into *Tg*81. *Tg*81 mice inoculated with brain homogenate from mouse “g” developed prion disease with a median survival time of 91 days, whereas for the other brain homogenates a median survival time was not reached.

We then sought to assess whether these mice showed any alteration of prion susceptibility after PrP^Sc^ infection. After intracerebral (i.c.) administration of 3 x 10^5^ ID_50_ units of prions (Rocky Mountain Laboratory strain, passage 6, henceforth denoted RML), both wt and *Tg*(MoPrP^169F^) mice developed signs of scrapie with similar latencies (Fig 2A), whereas *Prnp*^-/-^ mice died of intercurrent disease without developing scrapie. Reinoculation of 1% brain homogenates from prion-infected wt and *Tg*(MoPrP^169F^) mice into *Tg*a20 mice, which express 10 fold higher amounts of PrP than C57BL/6 mice [23], again resulted in scrapie (Fig 2C and S3A Fig).

We next sought to determine whether the MoPrP^169F^ mutation could have an influence on the species barrier. After intracerebral inoculation with hamster-adapted prions (strain 263K [26]; 3 x 10^6^ ID_50_ units), mice failed to develop signs of disease within 500 days of observation (Fig 2B). However, brain homogenates from these animals inoculated into *Tg*81 mice overexpressing Syrian hamster *Prnp* [27] occasionally led to prion disease and death, similarly to what was reported for wt mice [26] (Fig 2D and S3B Fig).

We next asked whether the MoPrP^169F^ mutation would have any effect on antibody-mediated neurotoxicity [28,29]. We therefore injected the monovalent fragment Fab_1_ of the anti-prion antibody POM1 into the brains of *Tg*(MoPrP^169F^);*Prnp*^-/-^ and C57BL/6 wt mice *(*S4 Fig). Transgenic and wt mice showed similar neurotoxic effects, which could be suppressed by pre-incubating Fab_1_-POM1 with a 5-fold molar excess of recombinant murine PrP fragment encompassing residues 90–231.

### PrP^Sc^ of RML infected *Tg*(MoPrP^169F^);*Prnp*^-/-^ mice is protease sensitive

**Fig 3:**
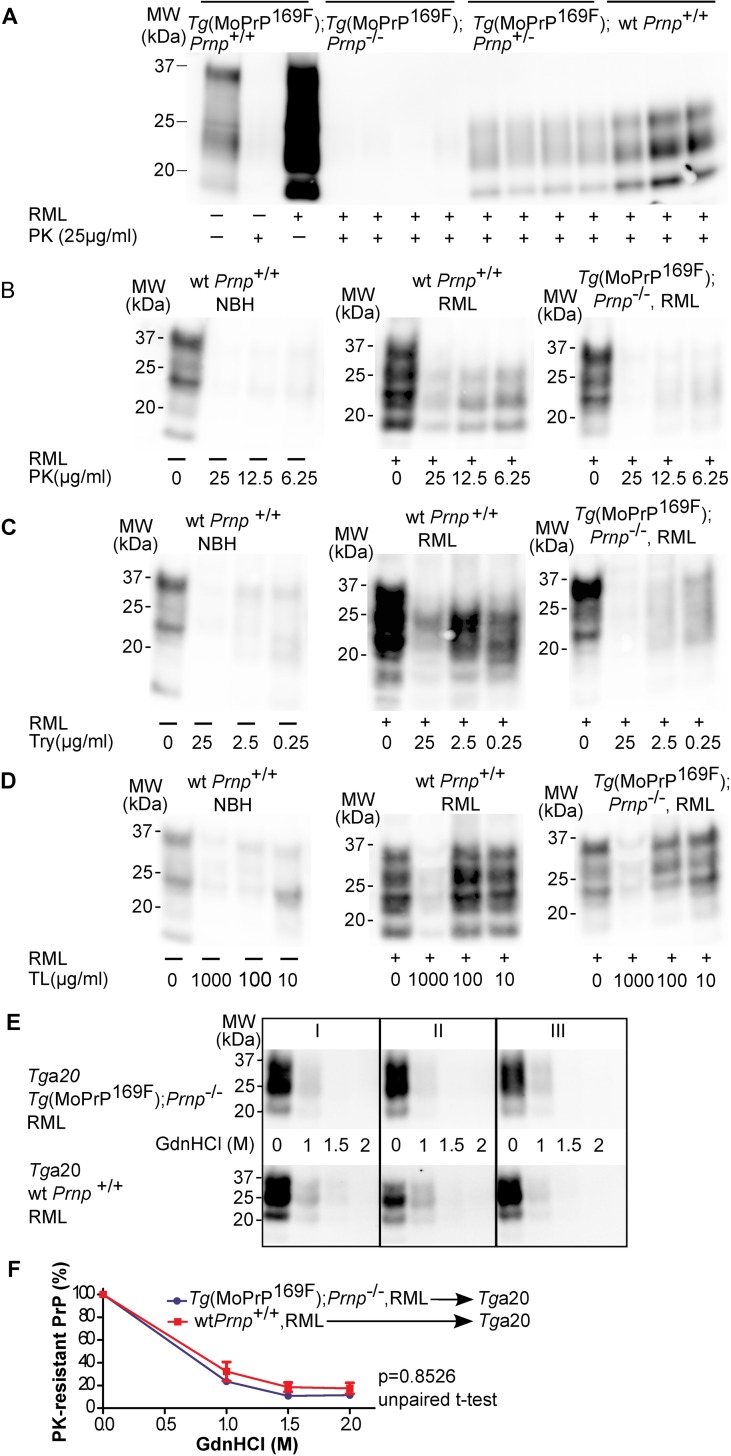
RML-inoculated *Tg*(MoPrP^169F^);*Prnp*^*-/-*^ mice accumulate protease-sensitive PrP^Sc^ in the brain. (A) Western blot analysis of brain homogenates from RML-inoculated *Tg*(MoPrP^169F^) mice showed increased sensitivity to PK digestion (25 μg/ml PK for 30 min at 37°C) compared to wt *Prnp*^+/+^ mice. (B) A gradient of different PK concentrations showed that PrP^Sc^ in the brain homogenates from *Tg*(MoPrP^169F^);*Prnp*^*-/-*^ was sensitive to PK concentrations up to 6.25 μg/ml. (C) Same as (B) for trypsin (Try) digestion. Brain homogenates (10%) from *Tg*(MoPrP^169F^);*Prnp*^-/-^ mice showed a reduction of PrP signal after trypsin treatment up to a concentration of 2.5μg/ml. (D) Same as (B) for thermolysin (TL) digestion. Thermolysin showed no significant effect on PrP^Sc^ in the brain homogenate from *Tg*(MoPrP^169F^);*Prnp*^-/-^ at a concentration of 100μg/ml (70°C, 30 min), which is in contrast to 10% brain homogenate from wild-type *Prnp*^+/+^ mice inoculated with NBH, where no PrP could be detected at this concentration. (E) Western blot analysis of brain homogenates from *Tg*a20 mice infected with prions from *Tg*(MoPrP^169F^);*Prnp*^*-/-*^ or wt mice. Homogenates were treated with increasing GdnHCl concentrations and PK-digested. (F) Quantitative analysis of the Western blot data shown in panel E. Each data point is the mean of three biological replicates ± SD. WT and PrP^169F^ prions showed similar GdnHCl stability (p = 0.8526, unpaired t-test). The antibody POM1 was used for detection. Molecular sizes are indicated in kDa. NBH: noninfectious brain homogenate.

Treatment of brain homogenates from RML-infected, approximately 1-year-old, terminally sick *Tg*(MoPrP^169F^);*Prnp*^-/-^ mice with PK (25 μg/ml, 37°C, 30 min) led to almost complete loss of PrP immunoreactivity in Western blots (Fig 3A and S10A Fig), indicating that very little or no PrP^res^ was formed. Further assessment of PK sensitivity with decreasing PK concentrations showed that PrP^res^ from *Tg*(MoPrP^169F^);*Prnp*^-/-^ mice was sensitive to PK treatment down to a concentration of 6.25 μg/ml (Fig 3B and S10B Fig). In contrast, homogenates of terminally scrapie-sick wt mice showed PrP^res^ even after treatment with a 20-fold higher PK concentration (S5 Fig and S13 Fig). To assess the presence of shorter PK-resistant fragments that might go undetected by POM1, we also probed the Western blots with a panel of additional anti-PrP antibodies (POM3, POM5, POM6, POM15 and POM19) targeting different regions of the PrP molecule [28]. All antibodies recognized similar differences in PK sensitivity between RML infected wt- and *Tg*(MoPrP169F);*Prnp*^*-*/-^ animals (S6 Fig and S14 Fig) and no alternative bands. *Tg*(MoPrP^169F^);*Prnp*^+/-^ mice showed reduced levels of PrP^res^ compared to wt animals, yet showed the same pattern of enhanced electrophoretic motility resulting from N-terminal proteolysis and the typical triplet bands corresponding to unglycosylated, monoglycosylated, and diglycosylated isoforms (Fig 3, S7 Fig, S10 Fig and S15 Fig). Trypsin treatment also resulted in a major reduction of signal in transgenic mice but not in wt PrP^C^ expressing animals (Fig 3C and S10C Fig). However, thermolysin (TL) treated samples (100 μg/ml) retained PrP immunoreactivity (Fig 3D and S10D Fig), as published previously for PK sensitive PrP [30], albeit without the characteristic shift in gel mobility caused by PK.

To determine whether the increased PK sensitivity of MoPrP^169F^ prions was heritable (and hence indicative of a new prion strain), we passaged MoPrP^169F^ prions into *Tg*a20 mice and investigated the properties of the resulting prions in a conformational stability assay [31] (Fig 3E and S10E Fig). The GdnHCl denaturation curves showed no significant difference (p = 0.8526, unpaired t-test) between RML and MoPrP^169F^ prions passaged through *Tg*a20 mice (Fig 3E and 3F and S10E Fig). Crucially, infection of *Tg*a20 mice with MoPrP^169F^ prions caused the generation of PrP^Sc^ with PK resistance. Similar results were seen after passage of 263K-infected transgenic mice into hamster PrP-expressing *Tg*81 mice (S8 Fig and S16 Fig). Hence passage of PK-sensitive *Tg*(MoPrP^169F^);*Prnp*^-/-^ prions into *Tg*a20 or *Tg*81 mice led to reappearance of the original strain properties. PK sensitivity of MoPrP^169F^ prions is therefore a phenotype due to recruitment of host expressed PrP^C^ into PrP^Sc^, rather than a prion-encoded strain.

**Fig 4:**
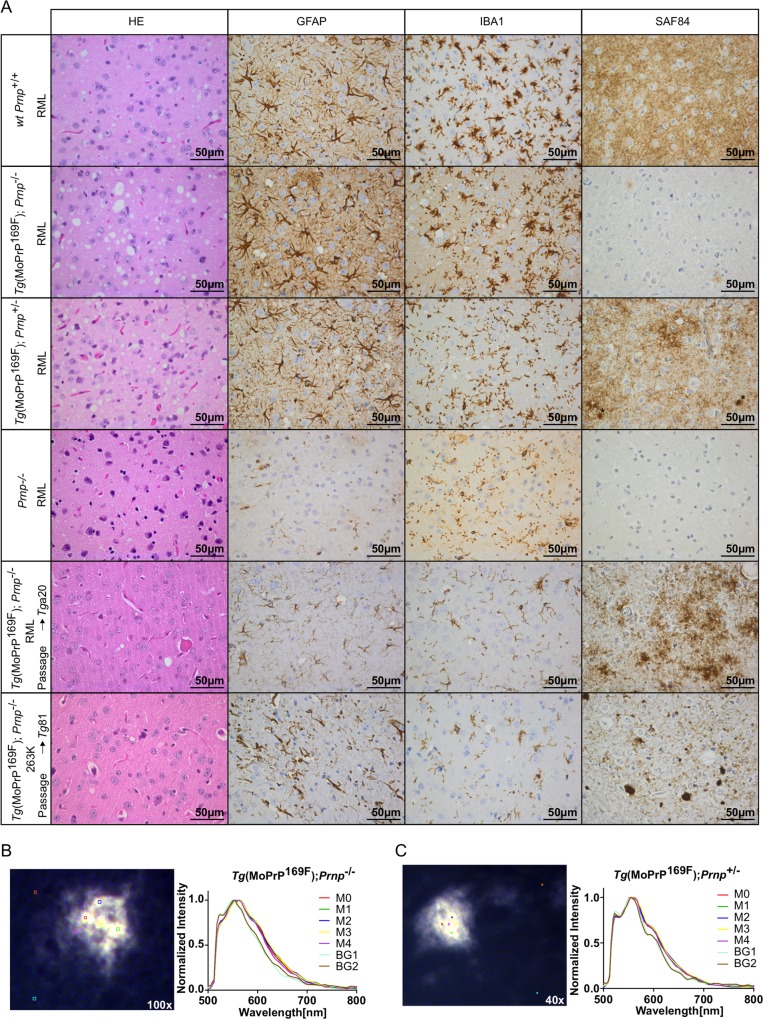
Histopathological patterns of prion disease and Lin5050 positive plaques in *Tg*(MoPrP^169F^) mice. **(A)** Brain sections from RML-infected *Tg*(MoPrP^169F^);*Prnp*^-/-^, *Tg*(MoPrP^169F^);*Prnp*^+/-^, *Prnp*^*-/-*^ and *Prnp*^+/+^ mice, as well as *Tg*a20 and *Tg81* mice inoculated with *Tg*(MoPrP^169F^);*Prnp*^-/-^ brain homogenates. Hallmarks of prion disease including vacuolation (hematoxylin and eosin; HE), astrogliosis (glial fibrillary acidic protein; GFAP) and microglial activation (activated microglial marker; IBA1), were detected in terminally sick *Tg*(MoPrP^169F^) and RML-infected wild-type *Prnp*^+/+^ mice. RML-infected wild-type (wt) mice showed the typical diffuse synaptic pattern of SAF84 positive signals (first row), and *Tg*(MoPrP^169F^);*Prnp*^+/-^ showed some plaque-like deposits (asterisk; third row). Few weakly stained PrP^Sc^ deposits were visible in *Tg*(MoPrP^169F^);*Prnp*^-/-^ and no PrP signals in *Prnp*^-/-^ brains (rows 2 and 4). After passage of *Tg*(MoPrP^169F^);*Prnp*^-/-^ brain homogenate into *Tg*a20 mice, synaptic and plaque-like PrP deposits were observed in the cortex (5^th^ row). Passage of 263K-infected *Tg*(MoPrP^169F^);*Prnp*^-/-^ into *Tg*81 mice resulted in a prion disease with PrP plaque formation in the cortex (6^th^ row). Scale bar: 50 μm. (B) Frozen section (10 μm) of *Tg*(MoPrP^169F^);*Prnp*^-/-^ brain showed LIN5050 stained aggregates. Fluorescence emission spectra were recorded in 5 regions (M0-M4) at 500–800 nm and compared to the surrounding tissue (M5-M6) (magnification 100x). (C) Same as (B) but from a *Tg*(MoPrP^169F^);*Prnp*^+/-^ mouse (magnification 40x).

Histological analyses of RML-infected mice (*Tg*(MoPrP^169F^);*Prnp*^-/-^ (n = 7), *Tg*(MoPrP^169F^);*Prnp*^+/-^ (n = 9); wt *Prnp*^+/+^ (n = 6)) consistently showed vacuolated brains with severe astrogliosis (GFAP) and microglial activation (Fig 4A). Immunohistochemistry for PrP^Sc^ (with antibody SAF84 upon antigen retrieval with protease 2 (Roche, Ventana medical systems) for 16 min) showed few weakly stained plaque-like PrP deposits in *Tg*(MoPrP^169F^);*Prnp*^-/-^ animals, whereas the typical synaptic pattern of PrP was observed in mice expressing wt PrP^C^. Occasionally stronger signals of more compact SAF84-positive plaque-like deposits (asterisk) could be observed in *Tg*(MoPrP^169F^);*Prnp*^+/-^ mice (Fig 4A). The passage into *Tg*81 and *Tg*a20 mice histologically confirmed the presence of PrP^Sc^ in the cortex. Frozen hippocampal sections of RML-infected *Tg*(MoPrP^169F^) and C57BL/6 mice were also stained with the luminescent conjugated polythiophene, LIN5050, which specifically detects amyloid aggregates [32,33]. In RML-infected MoPrP^169F^ transgenic mice (with or without endogenous PrP^C^ expression), small plaque-like LIN5050-positive deposits were observed (Fig 4B and 4C), and fluorescence emission spectra with an emission maximum E_max_ at a wavelength of 565nm were recorded. In RML-inoculated C57BL/6 mice, the synaptic pattern of PrP^Sc^ (detectable by SAF84 immunostaining) could not be visualized with LIN5050, which primarily stains plaques and plaque-like deposits [32], showing that RML-infected *Tg*(MoPrP^169F^) and C57BL/6 mice display different morphological patterns of PrP^Sc^ deposition.

### Decreased high-density aggregates in RML-inoculated *Tg*(MoPrP^169F^) mice

**Fig 5:**
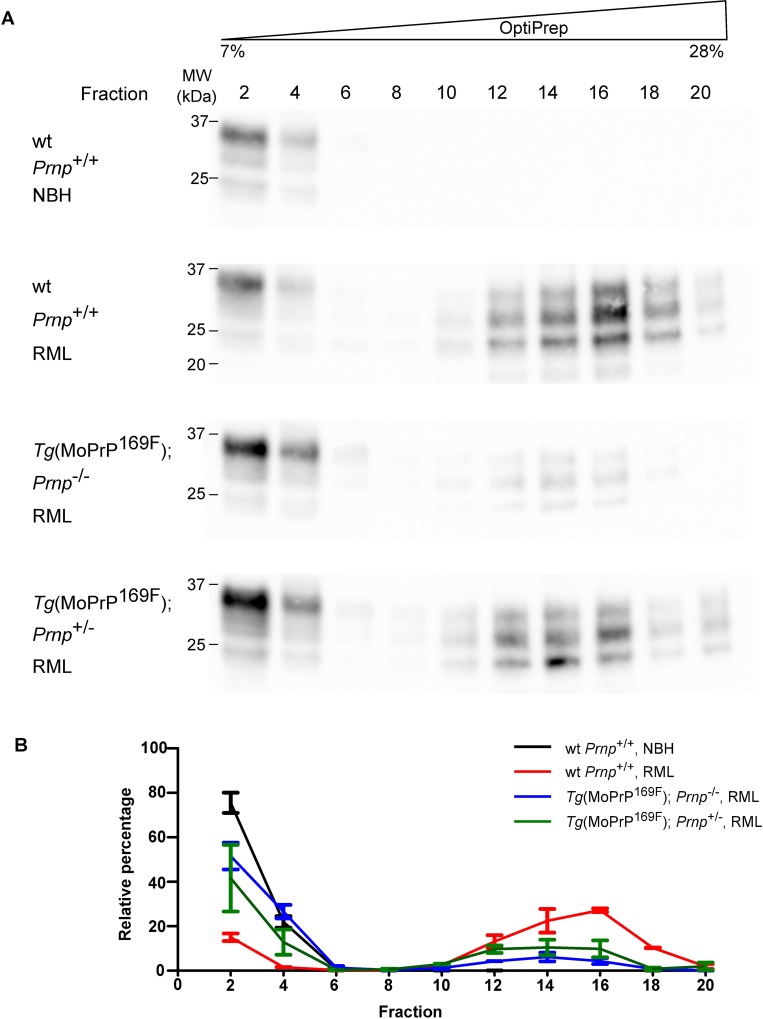
Brains of RML-infected *Tg*(MoPrP^169F^);*Prnp*^-/-^ harbor reduced amounts of high density PrP. (A) Western blot analysis of differentially fractionated brain homogenates from RML-infected *Tg*(MoPrP^169F^);*Prnp*^-/-^ and *Tg*(MoPrP^169F^);*Prnp*^+/-^ mice showed reduced signals of high density PrP aggregates compared to RML-infected wild-type (wt) mice (fractions 10 to 20; OptiPrep 7 to 28%). Wild-type *Prnp*^+/+^ mice inoculated with noninfectious brain homogenate (NBH) displayed only signals in the low density fractions (Fractions 2 and 4). Every second fraction was analyzed by Western blotting. PrP was detected by the anti-PrP antibody POM1. Molecular sizes are indicated in kDa. (B) Quantification of the PrP signal, using two technical duplicates per mouse (Western blots are shown in Fig 5A and S9 Fig; mean ± SEM).

To better understand the physical and morphological properties of the pathological MoPrP^169F^ in RML-infected mice, we analyzed brain homogenates by density gradient ultracentrifugation followed by Western blotting of each fraction [34]. In uninfected mice, most PrP was present in the low-density fractions (1–5), whereas all RML inoculated mice showed additional PrP signals in high-density fractions (10–20) (Fig 5A, S9A Fig, S11 Fig and S17 Fig). In contrast to RML-inoculated wt *Prnp*^+/+^ and *Tg*(MoPrP^169F^);*Prnp*^+/-^ mice, animals expressing only the transgenic PrP showed a strong reduction of high-density PrP aggregates. PK digestion confirmed the presence of PrP^res^ in the high-density fractions (S9B Fig and S18 Fig) and reduced amounts of PrP^res^ in *Tg*(MoPrP^169F^).

The relative quantity of PrP signals was calculated, with the highest amount of dense PrP deposits in RML-inoculated wt *Prnp*^+/+^ mice followed by transgenic mice with a hemizygous *Prnp* background (Fig 5B). In transgenic mice without endogenous PrP^C^ expression, only weak bands of PrP deposits in the high-density range could be detected. These findings indicate that *Tg*(MoPrP^169F^);*Prnp*^-/-^ mice contain reduced amounts of compact PrP aggregates in the brain.

## Discussion

One major goal of prion research is to better understand the relationship between PrP^Sc^ structure and pathogenesis. A powerful approach towards this goal relies on developing variants of PrP^C^ with well-defined structural features and molecular dynamics, and analyzing the consequences of their *in vivo* expression under conditions of health and disease. The design of such *in vivo* experiments is supported by the availability of atomic-resolution PrP^C^ structures [8–22]. Here we focused on the β2-α2 loop because its conformation affects cross-species transmission of prions and is even associated with the spontaneous generation of prions [35–37].

The replacement of tyrosine with phenylalanine at position 169 amounts to the removal of a single oxygen atom from the tyrosine residue. NMR studies showed that the conformation of the β2–α2 loop is unaffected by this amino acid exchange, whereas substitution by alanine or glycine results in a major rearrangement of the 3_10_-helical β2–α2 loop to a type-I β–turn conformation [20]. The Y169F mutation decreases the melting temperature by only 2.6°C [21], whereas for the variants with alanine or glycine at position 169 the melting temperature was lowered by ca. 10°C [21]. The substitution of Y169 by glycine in *Tg*(MoPrP^169,170,174^) [35] mice also prevented the development of the spontaneous TSE that was observed in *Tg*(MoPrP^170,174^) mice expressing the double mutation S170N and N174T[36,37]. *Tg*(MoPrP^169,170,174^) mice additionally showed a markedly enhanced transmission barrier [35], suggesting that residue 169 plays a crucial role in prion conversion, as was also demonstrated by *in vitro* conversion experiments [38].

After inoculation with two different prion strains, RML and 263K, mice expressing the Y169F mutant developed prion infectivity similarly to wt mice. Moreover, prions extracted from the brains of these mice were transmissible to *Tg*a20 and *Tg*81 mice, respectively. This indicates that the Y169F variant prion protein is fully competent to enable the acquisition and multiplication of prions as well as the transmission of *de novo* synthesized prions to additional hosts.

In addition to typical clinical signs of scrapie, infected Y169F mice showed neuronal vacuolation and extensive astrogliosis similar to wt mice, and RML-infected mice developed polythiophene-stainable plaques [32]. Moreover, injection with Fab_1_-POM1 induced similar toxicity in Y169F transgenic mice and wt mice. Finally, expression of MoPrP^169F^ suppressed the progressive demyelination seen in *Prnp*^-/-^ mice [25]. We conclude that both the physiological and pathogenic properties of MoPrP^169F^ are indistinguishable from those of wt MoPrP.

**Fig 6:**
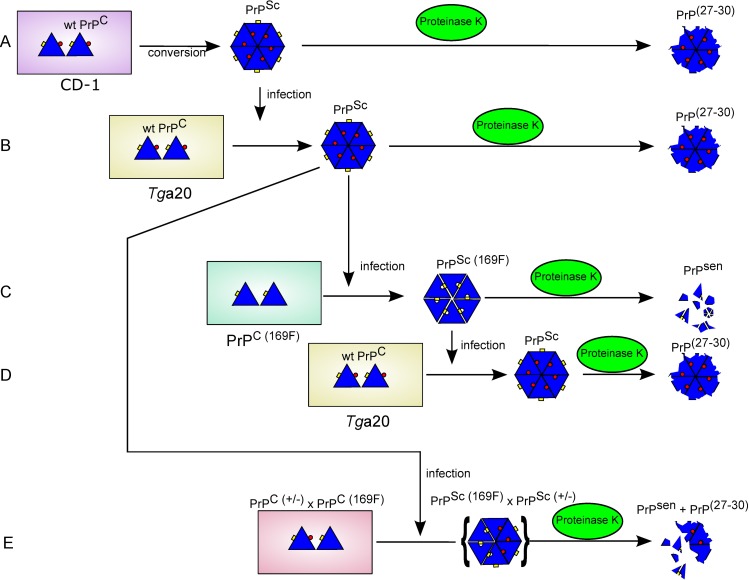
Schematic presentation of a rationale for the observed propagation, or absence thereof, of MoPrP^169F^-related PK sensitivity. Triangles: PrP; red circle: oxygen atom of Y169; yellow rectangle: aggregation interface of PrP^Sc^. **A**: Exposure of wild-type (wt) mice to RML prions catalyzes the templated conversion of PrP^C^ into PrP^Sc^. The hydroxyl group of Y169 participates in the formation of tightly packed PrP^Sc^ aggregates that form a PK-resistant core (PrP^27–30^). **B**: Infection of mice overexpressing wt PrP^C^ again induces infectious PK-resistant PrP^Sc^ aggregates, which results from recruiting wt PrP^C^ of the host into PrP^Sc^. **C**: The Y169F mutation impairs the packing of PrP^Sc(169F)^ molecules. This leads to infectious, yet PK-sensitive, PrP aggregates. **D**: Transmission of PrP^169F^ prions to mice expressing wt PrP^C^ results again in tightly packed aggregates, which form PrP^27–30^ after PK digestion. **E**: In mice co-expressing both wt PrP^C^ and PrP^C(169F)^, prion infection leads to reduced amounts of PK resistant PrP^Sc^, indicative of hybrid PrP/PrP^169F^ aggregates or of coexisting loosely and tightly packed PrP^Sc^ aggregates.

Despite their unabated pathogenicity, MoPrP^169F^ prions were surprisingly sensitive to PK and trypsin, and were less dense/smaller when compared to RML prions. Rather than representing a strain shift, these traits were host-encoded, and the original strain properties of RML re-emerged after passage of MoPrP^169F^ into *Tg*a20 mice (Fig 6). Furthermore, co-expression of PrP^169F^ did not conspicuously interfere with the conversion of wt PrP^C^ into PrP^Sc^. This is in contrast to other PrP variants which often exert a dominant-negative effect on prion propagation [39–41]. Therefore, a subtle structural variation in the β2–α2 loop of PrP^C^ affected sensitivity of PrP^Sc^ to proteases and reduced the amounts of large/compact aggregates arising after prion infection. Although the overall structure of monomeric MoPrP^169F^ is similar to that of wt PrP^C^, the Y169F mutation causes the loss of the side-chain hydrogen bond formed between Tyr169 and Asp178, which stabilizes the π-stacking interactions formed between the aromatic residues Tyr169, Phe175, and Tyr218 [21,42,43]. MD simulations for PrPs carrying pathological mutations demonstrated that a loss of the π-stacking interactions could lead to a rearrangement of residue 169 to a more solvent exposed trans conformation, which affects the distance between the β2–α2 loop and α3 helix and leads to a more flexible α3 helix [21,42,43]. Further simulations also showed that this effect can be determined by the presence of either methionine or valine at position 129, as Tyr128 forms a hydrogen bond with Asp178, which can be affected by the kind of amino acid at position 129 [43]. Hence, a plausible explanation might be that a modulation of this network in PrP^C^ also affects the aggregational PrP^Sc^ states, leading to looser packing and increased PK sensitivity of MoPrP^169F^ aggregates. This is also in agreement with the feature of MoPrP^170,174^ prions being mostly PK-sensitive after passaging them into *Tg*a20 mice [36], as the conformation and dynamics induced by residue 170 also plays a crucial role in the preservation of this network [44].

The physical basis of PK resistance is likely to reside in steric crowding, which would limit contacts with solvent water and the protease. The removal of the hydroxyl group at residue 169 could thus ease the accessibility of water and proteolytic enzymes, and loss of packing density resulting from the elimination of the–OH group could provide a rationale for the results of density-gradient centrifugation (S9A Fig and S17 Fig). The reduced density of MoPrP^169F^ prions is also in agreement with other studies, which demonstrated that PK-sensitive forms of PrP^Sc^ are found in lower-density fractions. This could also be rationalized by the assumption that PK-sensitive forms consist of smaller aggregates of PrP molecules [45–47]. Our findings thus yield another example of a prion disease that generates infective PK-sensitive aggregates, as was previously seen in several familial human prion diseases and related mouse models [37,48–52].

In conclusion, our findings demonstrate that a subtle change in the prion protein covalent structure, i.e. the removal of a single oxygen atom, has a significant effect on a physicochemical property of PrP^Sc^ without abolishing its infectivity. Further studies using structurally well-defined PrP variants may yield direct insights into prion generation, thereby advancing the development of novel diagnostic and therapeutic procedures.

## Materials and methods

### Generation of *Tg(*MoPrP^169F^) mice

Half-genomic PrP vector was genetically modified to exchange tyrosine of PrPY169 against phenylalanine by using the Quick change lightning site-directed mutagenesis kit (Stratagene) and the primer set fwd: AGG CCA GTG GAT CAG TTC AGC AAC CAG AAC AAC. Rev: GTT GTT CTG GTT GCT GAA CTG ATC CAC TGG CCT. PCR was performed according to the company´s protocol. The linearized transgenic construct (NOT1/ Sal1) was injected into 296 fertilized *Prnp*^+/+^ (B6D2F1) oocytes, resulting into four transgene positive and 15 transgene negative mice that reached weaning age. The generated transgene positive mice were backcrossed 3 times with *Prnp*^0/0^ (B6.129-Prnp<tm1ZH1>) and maintained in a hemizygous transgenic background.

### Quantification of transgene expression using Förster Resonance Energy Transfer (FRET)

A FRET based assay was established for the purpose of quantifying transgene expression by measuring PrP levels of 10% brain homogenates from *Tg*(MoPrP^169F^); *Prnp*^-/-^, wt *Prnp*^+/+^, wt *Prnp*^+/-^ and *Prnp*^-/-^ mice. Europium (Eu^3+^) donor and allophycocyanin (APC) acceptor fluorophores were coupled to anti-PrP holoantibodies POM1 and POM2 recognizing the globular domain and the octarepeats, respectively. The donor POM1-Eu^3+^ conjugate is excited at wavelength 340 nm and transfers energy to the acceptor conjugate POM2-APC when the distance between acceptor and donor is <10 nm. POM2-APC then emits light at wavelength 665 nm, which can be measured with a suitable time-resolving spectrofluorimeter. For PrP level detection in homogenates, the Eu^2+^-POM1 and APC-POM2 antibody pair was added, measured immediately using a FRET reader and normalized to total protein.

### Animal experimentation, animal welfare and ethics statement

All animal experiments were conducted in strict accordance with the Rules and Regulations for the Protection of Animal Rights (Tierschutzgesetz and Tierschutzverordnung) of the Swiss Bundesamt für Lebensmittelsicherheit und Veterinärwesen BLV. All animal protocols and experiments performed were specifically approved for this study by the responsible institutional animal care committee, namely the Animal Welfare Committee of the Canton of Zurich (permit numbers 41/2012). All efforts were made to minimize animal discomfort and suffering.

### Prion inoculation

Transgenic mice (F3), *Prnp*^-/-^ mice or B6 *Prnp*^+/+^ mice were anaesthetized using isofluorane and inoculated with 30μl of 0.1% brain homogenate, diluted in sterile PBS/ 5% BSA, from Rocky Mountain Laboratory infected CD1 (3x10^5^ ID_50_ units) or 263K (3x10^6^ ID_50_ units) injected SHaPrP mice (*Tg*81). Inoculated mice were monitored every other day and actions were taken to minimize animal suffering and distress as indicated in S1 Table. To euthanize mice CO_2_ inhalation was used on the day of appearance of terminal clinical signs of scrapie. For confirmation of infectivity, the brains of inoculated mice were homogenized and heat inactivated for 25 min at 80°C. Heat inactivated and non-heat inactivated brain homogenates were diluted in PBS/ 5% BSA to a final concentration of 1%wt/vol. and injected (30μl) into *Tg*a20 mice (for RML inoculated animals) or *Tg*81 mice (for 263K treated transgenic mice).

### Sample preparation

Terminally sick mice were sacrificed and organs were taken. The brain was sectioned sagittally, and one hemisphere was fixed in 4% formalin. A coronal section of brain containing hippocampus was placed in HANKS buffer and the rest was snap frozen in liquid nitrogen. Brain homogenates from snap frozen samples (10% wt/vol. in 0.32M sucrose diluted in PBS) were used for further analyses.

### Proteolytic digestion with PK, thermolysin, or trypsin

Brain homogenates (10 or 20 μg in 20 μl) were optionally treated with a final concentration of 25, 12.5 or 6.25μg/ml of trypsin or proteinase-K (37°C), or 1000, 100 or 10 μg/ml of thermolysin (70°C) for 30 min. Samples were subsequently mixed with 4x loading dye and denatured at 95°C for 5 min. Fifteen microliters of each sample were separated on a 4–12% Bis-Tris SDS polyacrylamide gel (NuPAGE, Invitrogen) and electrotransferred to nitrocellulose membranes. Membranes were blocked for 1 hour in 5% Topblock (Fluka) in Tris-buffered saline supplemented with Tween 20 [150 mM NaCl, 10 mM Tris-HCl, 0.05% Tween 20 (v/v)] and incubated overnight with anti-PrP antibodies POM1, POM3, POM5, POM6, POM15 or POM19 (200 ng/ml). As secondary antibody, horseradish peroxidase-conjugated goat anti mouse IgG (H+L) (1:10.000, Invitrogen) was used and immunoreactivity was visualized using chemiluminescence (Luminata crescendo, Merck Millipore).

### Conformational stability assay

Brain homogenates (10% w/v) from *Tg*a20 mice inoculated with brain homogenates from RML infected B6 wt *Prnp*^+/+^ or *Tg*(MoPrP^169F^);*Prnp*^-/-^ mice were dissolved in equal volumes of solubilization buffer (4% sarcosyl, 100 mM Tris HCl, pH7.4,) and incubated for 1h at 37°C. Aliquots of 20 μl were treated with 20 μl of GdnHCl solutions at final concentrations ranging from 0 to 2 M and incubated for 1h at 37°C. Final GdnHCl concentration was adjusted to 0.4 M in equal volumes. Samples were treated with PK at a final concentration of 25μg/ml for 30min at 37°C. Protease activity was blocked by adding 50% volume of protease inhibitor cocktail (complete Mini, Roche) dissolved in 10ml deionized H_2_O. A fourfold excess of methanol for protein precipitation was added and incubated overnight at -40°C. Samples were centrifuged for 1h at 20.000 g and 4°C. The pellets were resuspended in 4x loading dye (NuPAGE LDS sample buffer, Thermo Fisher Scientific) and analyzed on a 4–12% Bis-Tris SDS polyacrylamide gel (NuPAGE, Invitrogen) followed by Western blotting as described above.

### Protein density gradient analysis

For each tested sample, 400 μg of brain homogenate diluted in PBS was solubilized in an equal volume of solubilization buffer [50 mM HEPES (pH 7.4), 300 mM NaCl, 10 mM EDTA, 2 mM dithiothreitol (DTT), 4% (wt/vol) dodecyl-d-maltoside (Sigma)] and incubated for 45 min on ice. Sarkosyl (N-lauryl sarcosine; Fluka) was added to a final concentration of 2% (wt/vol), followed by additional 30 min incubation on ice. Four hundred microliters of the samples were loaded on a 3.6 ml continuous 7 to 28% density gradient (OptiPrep, Sigma), with a final concentration of 25 mM HEPES (pH 7.4), 150 mM NaCl, 2 mM EDTA, 1 mM DTT, and 0.5% sarkosyl. Samples were centrifuged at 52,000 rpm for 90 min at 4°C (with a Discovery M150SE Micro-Ultracentrifuge and S52-ST swinging bucket). Fractions of 200 μl were collected and 20 μl per fraction was analyzed on 4–12% Bis-Tris SDS polyacrylamide gel (NuPAGE, Invitrogen) followed by Western blotting as described above. The signal was quantified with the freeware analysis program image studio lite (Li-cor).

### Histological analyses

Formalin fixed brain hemispheres were decontaminated in formic acid for 60 min to eliminate prion infectivity. After additional fixation in formalin, the tissue was paraffin embedded. Paraffin sections (3 μm) were stained with H&E. Immunohistochemical stainings for GFAP (1:13000 DAKO) and IBA1 (1:1000) were performed using standard methods. For SAF84 immunostains, sections were incubated in 98% formic acid for an additional 6 min after deparaffinization and washed in distilled water for 30 min. Sections were treated with citrate buffer (pH 6.0) for 3 min at 100°C. After adapting to room temperature, sections were incubated in Ventana buffer, and stained with the NEXEX immunohistochemistry robot (Ventana Instruments) using an iVIEW DAB Detection Kit (Ventana). After incubation with protease 2 (Ventana) for 16 min, sections were incubated with anti-PrP SAF-84 (SPI-Bio, A03208, 1:200) for 32 min and counterstained with hematoxylin.

### LCP staining on frozen sections

Brain samples, frozen in HANKS buffered salt solution, were cut (10 μm) and dried for 1h at room temperature. Tissue was fixed on slides with 100% ethanol for 10 min and subsequently washed in deionized water. Slides were preincubated for 10 min in PBS followed by staining with LIN5050 diluted in PBS ([4 μM] final concentration) for 30 min at room temperature. Sections were embedded with fluorescent mounting medium (DAKO) and stored at 4°C.

### Nerve fibre preparations

Sciatic nerves were fixed in 2% glutaraldehyde supplemented with 0.1 M sodium phosphate buffer at pH 7.4 and processed following standard procedures. At least 8 axons per nerve were teased on a glycerine gelatine covered glass slide.

#### Structural image

The structural representation in Fig 1A was created using PyMOL (the PyMOL Molecular Graphics System Version 1.8 Schrödinger, LLC; http://pymol.sourceforge.net/faq.html).

## Supporting information

S1 Fig PrP expression level by Western blot. Western blot analysis of *Tg*(MoPrP^169F^);*Prnp*^-/-^ expression reveals PrP levels comparable to wild-type (wt) *Prnp*^+/+^ mice. For *Tg*(MoPrP^169F^) each lane represents a biological replicate. PrP was stained with the anti-PrP antibody POM1 (200ng/ml). Actin was stained as loading control (1:10000). Quantitative analysis of these samples was performed by FRET assay as shown in Fig 1B.
(TIF)

S2 Fig Survival chart of *Tg*(MoPrP^169F^);*Prnp*^-/-^, *Prnp*^-/-^ and *Tg*(MoPrP^169F^);*Prnp*^+/-^ mice. The median survival of *Tg*(MoPrP^169F^);*Prnp*^-/-^ (767.5 days), *Prnp*^-/-^ (663.5 days) or *Tg*(MoPrP^169F^);*Prnp*^+/-^ (801 days) mice failed to show any significant difference.
(TIF)

S3 Fig Survival chart of passaged RML or 263K inoculated mice without heat-inactivation of the inoculum. In (A) 1% brain homogenate from RML inoculated mice was intracerebrally injected (30μl) into *Tg*a20 mice (mouse “a” and “b” correspond to *Tg*(MoPrP^169F^);*Prnp*^-/-^, mouse “c” to *Tg*(MoPrP^169F^);*Prnp*^+/-^ and mouse “d” to wt *Prnp*^+/+^ (see Fig 2A). (B) 1% brain homogenate from *Tg*(MoPrP^169F^);*Prnp*^-/-^ (mouse e, f and g; see Fig 2B) and *Prnp*^-/-^ (mouse h; see Fig 2B) inoculated with 263K were intracerebrally passaged into *Tg*81 mice. Heat inactivated noninfectious brain homogenate from *Tg*(MoPrP^169F^);*Prnp*^+/-^ or *Tg(*MoPrP^169F^);*Prnp*^-/-^ mice was used as control.
(TIF)

S4 Fig Fab_1_ POM1 injection into the brains of wt *Prnp*^+/+^ and *Tg*(MoPrP^169F^);*Prnp*^-/-^ mice. As expected, injection of 8μg of Fab_1_ POM1 into the left hemisphere of wt *Prnp*^+/+^ mice or *Tg*(MoPrP^169F^);*Prnp*^-/-^ mice resulted in lesions detected by MRI (4.7 tesla) without significant differences between the two groups. The effect could be blocked by preincubation of Fab_1_ POM1 with 5 molar excess of the recombinant murine PrP fragment (encompassing residues 90–231) diluted in PBS before administration into the brain. Each dot represents a biological replicate. Data are presented as mean ± SD.
(TIF)

S5 Fig Proteolytic digestion with PK. Brain homogenates (20 μg total protein per well) from RML infected B6 *Prnp*^*+/+*^, *Tg*(MoPrP^169F^);*Prnp*^+/-^ or *Tg*(MoPrP^169F^);*Prnp*^*-/-*^ mice were treated with concentrations of proteinase K ranging from 25 to 200 μg/ml for 30 min at 37°C. Samples were subsequently mixed with 4x loading dye (NuPAGE, invitrogen), denatured at 95°C for 5 min, separated on a 4–12% Bis-Tris SDS polyacrylamide gel and blotted onto a nitrocellulose membrane. Membranes were blocked for 1 h in 5% Topblock (Fluka) diluted in Tris-buffered saline supplemented with Tween 20 [150 mM NaCl, 10 mM Tris-HCl, 0.05% Tween 20 (v/v)] and incubated overnight at 4°C with anti PrP antibody POM1 (200 ng/ml). Horseradish peroxidase conjugated goat anti mouse IgG (H+L) (1:10.000, Invitrogen) was used as the secondary antibody and immunoreactivity was visualized using chemiluminescence (Luminata crescendo, Merck Millipore).
(TIF)

S6 Fig PrP detection using a panel of anti PrP antibodies. PK Western blot analysis of brain homogenates from RML-infected *Tg*(MoPrP^169F^);*Prnp*^-/-^ mice probed with a panel of different anti-PrP antibodies (POMs), covering different epitopes of PrP, compared to brain homogenates from RML-infected wt *Prnp*^+/+^ mice. All antibodies detected increased sensitivity to PK digestion and no shorter PK-fragments for MoPrP^169F^ prions. POM5 which recognizes the β2-α2 loop of PrP (residues 168–174) was not able to detect MoPrP^169F^, because of the point mutation in its recognition site. Brain homogenates from RML-infected wt *Prnp*^+/+^ mice were used as controls. 10 μg of total protein was treated with 25 μg/ml PK for 30 min at 37°C and loaded onto the gel. Non-digested samples from RML-infected wt and *Tg*(MoPrP^169F^);*Prnp*^-/-^ were used as controls. Bands were detected with the different anti-PrP antibodies as indicated in the Figure at a concentration of 200 ng/ml.
(TIF)

S7 Fig PrP detection using anti PrP antibody SAF84. Western blot analysis of 10% brain homogenates (10 μg total protein per well) from wt *Prnp*^+/+^, *Tg*(MoPrP^169F^);*Prnp*^+/-^ and *Tg*(MoPrP^169F^);*Prnp*^-/-^ mice treated with or without RML. Bands in the PrP region differed from the *Prnp*^-/-^ control. PrP was stained with the anti-PrP antibody SAF84 (1:1000). Actin is depicted as loading control (1:10000).
(TIF)

S8 Fig Passage of 263K inoculated mice into *Tg*81 mice. Passage of brain homogenate from 263K inoculated *Tg*((MoPrP^169F^);*Prnp*^-/-^ mouse (g) (previously shown in Figs 2B and S3B) into hamster PrP expressing *Tg*81 mice led to death and accumulation of PK resistant PrP. In contrast *Tg*(MoPrP^169F^);*Prnp*^-/-^ mice showed marked reduction of PK resistant material. In *Tg*(MoPrPF^169F^);*Prnp*^+/-^ mice the amount of PK resistant material was also reduced compared to the passaged *Tg*81 mice, but showed some interindividual variability. 20 μg of total protein per lane was treated or not with 25 μg/ml PK for 30 min at 37°C. Bands were detected with the anti PrP antibody POM1 (200 ng/ml).
(TIF)

S9 Fig Fractionation- and PK resistance analysis of brain homogenates from prion infected mice. (A) Western blot analyses of total PrP from differentially fractionated brain homogenate samples of wt *Prnp*^+/+^ mice inoculated with noninfectious brain homogenate or RML and RML inoculated *Tg*(MoPrP^169F^);*Prnp*^-/-^ and *Tg*(MoPrP^169F^);*Prnp*^+/-^ mice to confirm data from Fig 5. A technical replicate of the data presented in Fig 5 is shown with all fractions (1–20) loaded to a SDS-PAGE from a 7–28% OptiPrep gradient. (B) Effect of proteinase K on PrP aggregates migrating in low and high density fractions. Western blot analysis of pooled low (fractions 1–4) and high (fractions 12–15) density fractions from the ultracentrifugation experiment show PK (2 μg/ml) sensitive PrP in the low-density fractions. In high density fractions, PK resistance is maintained in wt *Prnp*^+/+^, whereas reduced resistance is observed in *Tg*(MoPrP^169F^) mice. Fractions were methanol precipitated and adjusted to 190 ng of total protein per lane. PrP was detected using the anti-PrP antibody POM1 (200 ng/ml).
(TIF)

S10 Fig Uncropped Western blots of Fig 3 with size markers. (TIF)

S11 Fig Uncropped Western blots of Fig 5 with size markers. (TIF)

S12 Fig Uncropped Western blots of S1 Fig. with size markers.
(TIF)

S13 Fig Uncropped Western blots of S5 Fig. with size markers.
(TIF)

S14 Fig Uncropped Western blots of S6 Fig. with size markers.
(TIF)

S15 Fig Uncropped Western blots of S7 Fig. with size markers.
(TIF)

S16 Fig Uncropped Western blot of S8 Fig. with size markers.
(TIF)

S17 Fig Uncropped Western blots of S9A Fig. with size markers.
(TIF)

S18 Fig Uncropped Western blots of S9B Fig. with size markers.
(TIF)

S1 File Excel sheet containing data that led to Fig 1B, Fig 2, Fig 3F, Fig 4B and 4C, Fig 5B, S2 Fig, S3 Fig and S4 Fig. (XLSX)

S1 Table Clinical assessment and scoring of mice inoculated with prions. The mice were observed every other day after prion inoculation for clinical signs including gait, grooming, activity, rough hair coat, limb paresis and ataxia. Once the mice showed the first sign of scrapie (grade 1), they were monitored every day and wet food was supplied in the cage. When the mice reached score grade 2 that hampered the mice reaching the water bottle, they were euthanized by CO_2_ inhalation.
(DOCX)
