# Supplementary figures and images for: Protease resistance of infectious prions is suppressed by removal of a single atom in the cellular prion protein
Source: PLoS One. 2017 Feb 16;12(2):e0170503. doi: 10.1371/journal.pone.0170503 (PMC5313174; doi:10.1371/journal.pone.0170503)

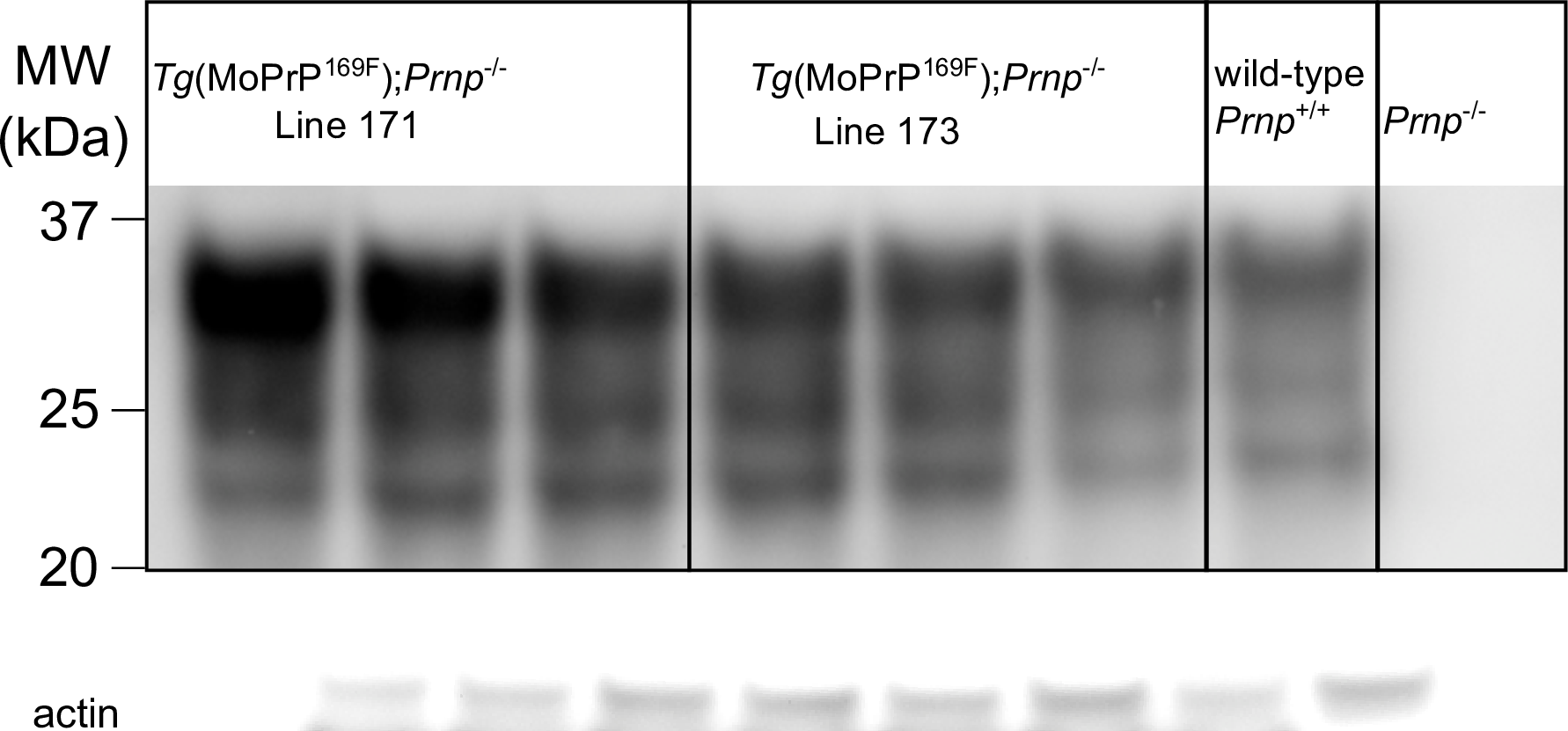

Supplement: S1 Fig — Western blot analysis of Tg(MoPrP169F);Prnp-/- expression reveals PrP levels comparable to wild-type (wt) Prnp+/+ mice. For Tg(MoPrP169F) each lane represents a biological replicate. PrP was stained with the anti-PrP antibody POM1 (200ng/ml). Actin was stained as loading control (1:10000). Quantitative analysis of these samples was performed by FRET assay as shown in Fig 1B. (TIF) [file pone.0170503.s001.tif]

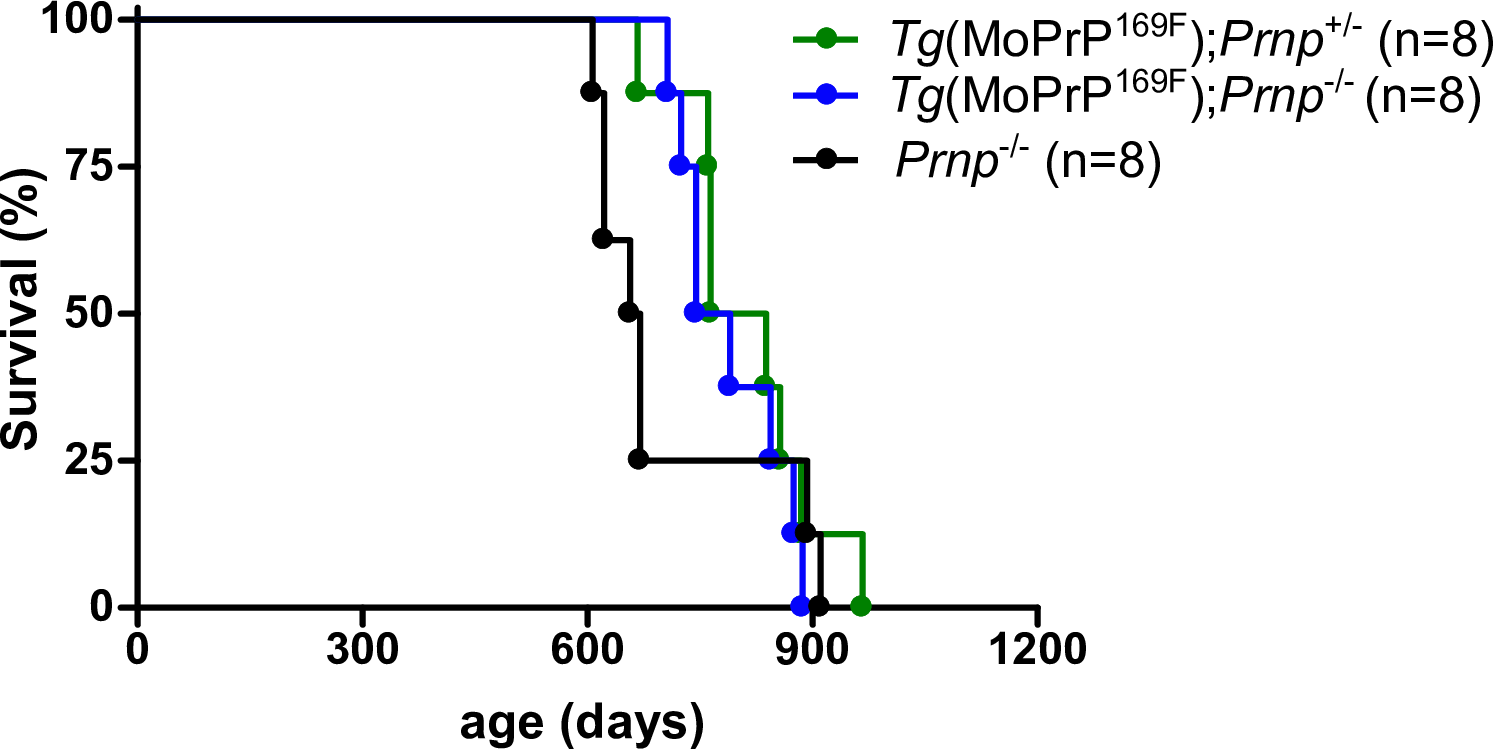

Supplement: S2 Fig — The median survival of Tg(MoPrP169F);Prnp-/- (767.5 days), Prnp-/- (663.5 days) or Tg(MoPrP169F);Prnp+/- (801 days) mice failed to show any significant difference. (TIF) [file pone.0170503.s002.tif]

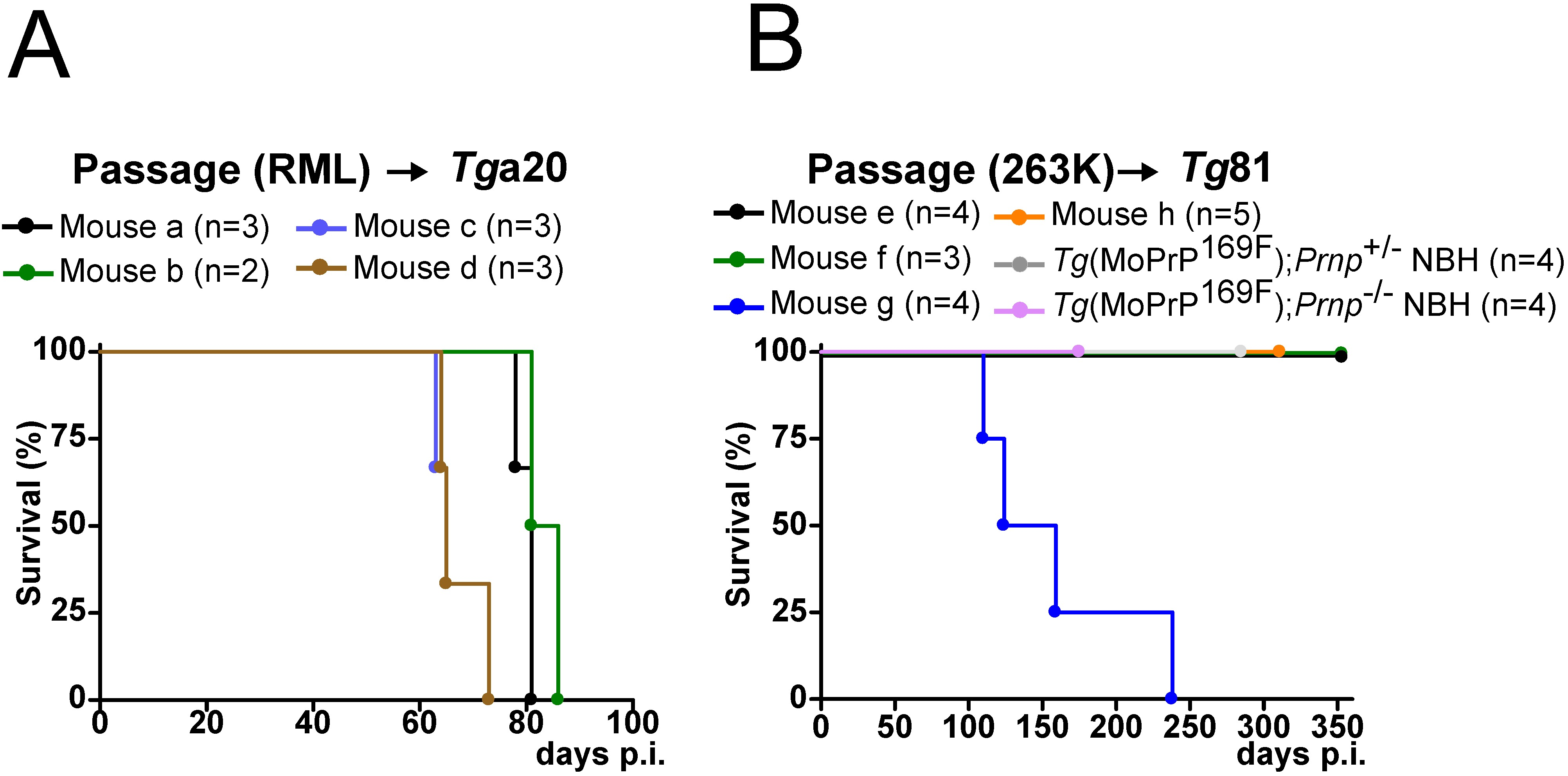

Supplement: S3 Fig — In (A) 1% brain homogenate from RML inoculated mice was intracerebrally injected (30μl) into Tga20 mice (mouse “a” and “b” correspond to Tg(MoPrP169F);Prnp-/-, mouse “c” to Tg(MoPrP169F);Prnp+/- and mouse “d” to wt Prnp+/+ (see Fig 2A). (B) 1% brain homogenate from Tg(MoPrP169F);Prnp-/- (mouse e, f and g; see Fig 2B) and Prnp-/- (mouse h; see Fig 2B) inoculated with 263K were intracerebrally passaged into Tg81 mice. Heat inactivated noninfectious brain homogenate from Tg(MoPrP169F);Prnp+/- or Tg(MoPrP169F);Prnp-/- mice was used as control. (TIF) [file pone.0170503.s003.tif]

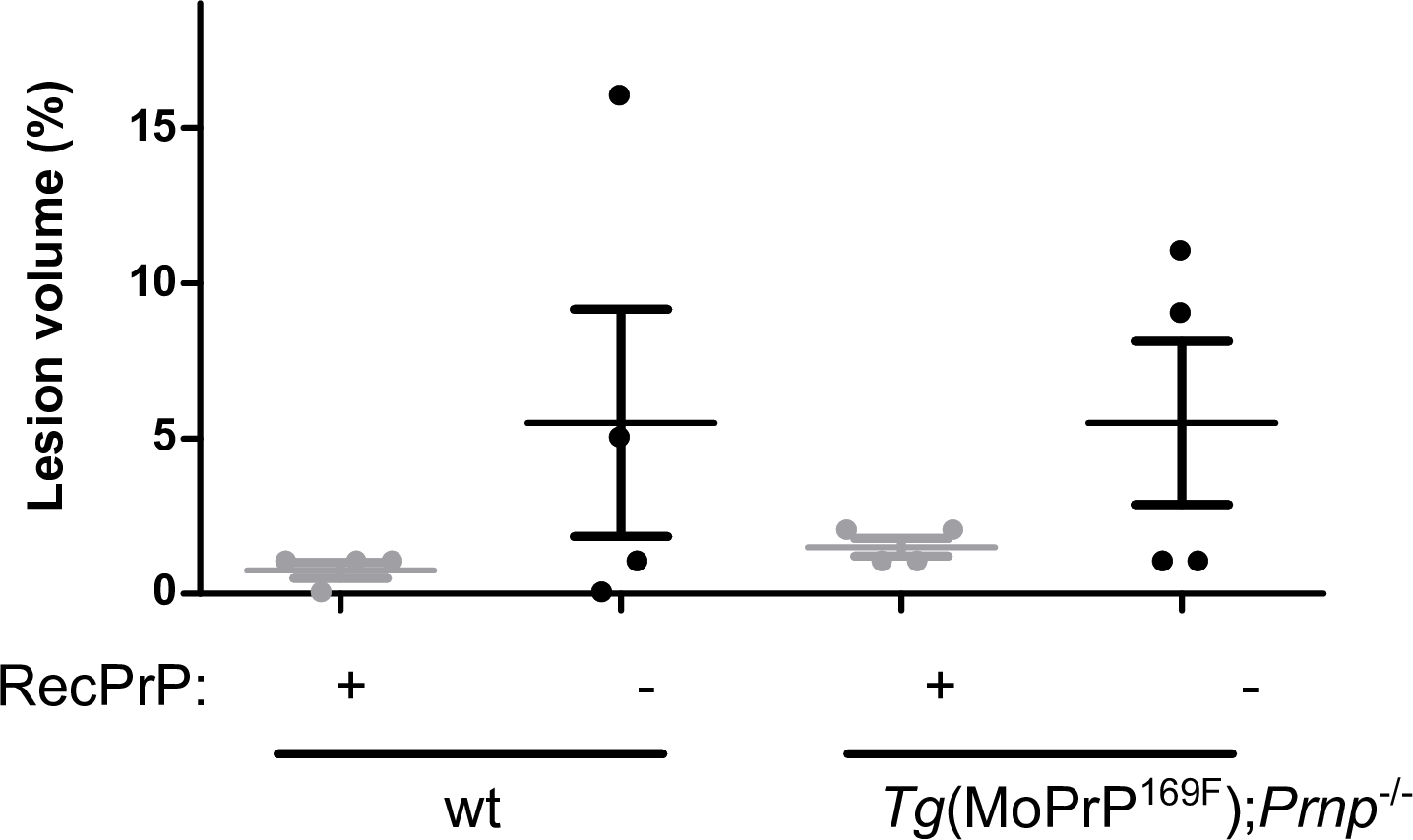

Supplement: S4 Fig — As expected, injection of 8μg of Fab1 POM1 into the left hemisphere of wt Prnp+/+ mice or Tg(MoPrP169F);Prnp-/- mice resulted in lesions detected by MRI (4.7 tesla) without significant differences between the two groups. The effect could be blocked by preincubation of Fab1 POM1 with 5 molar excess of the recombinant murine PrP fragment (encompassing residues 90–231) diluted in PBS before administration into the brain. Each dot represents a biological replicate. Data are presented as mean ± SD. (TIF) [file pone.0170503.s004.tif]

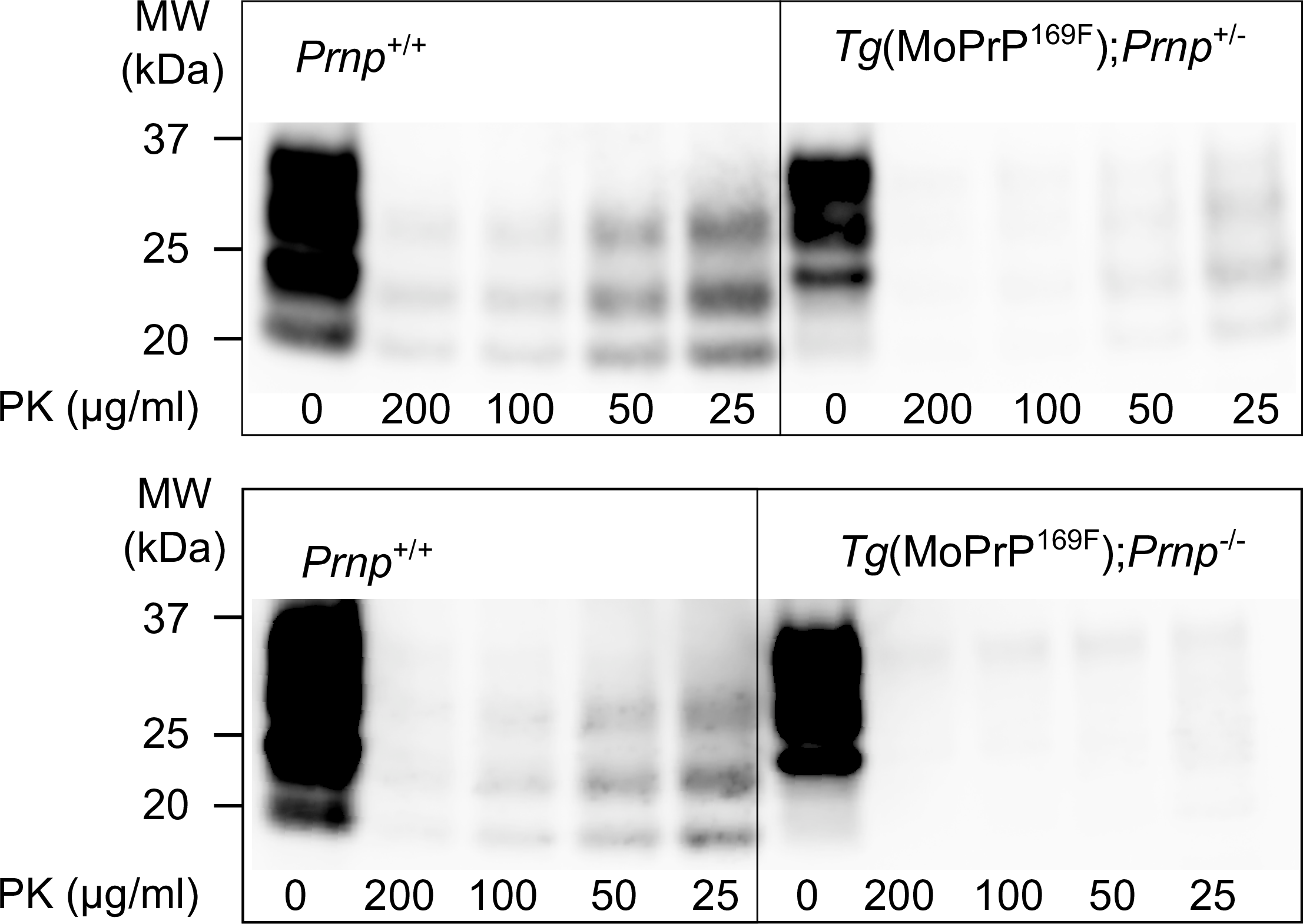

Supplement: S5 Fig — Brain homogenates (20 μg total protein per well) from RML infected B6 Prnp+/+, Tg(MoPrP169F);Prnp+/- or Tg(MoPrP169F);Prnp-/- mice were treated with concentrations of proteinase K ranging from 25 to 200 μg/ml for 30 min at 37°C. Samples were subsequently mixed with 4x loading dye (NuPAGE, invitrogen), denatured at 95°C for 5 min, separated on a 4–12% Bis-Tris SDS polyacrylamide gel and blotted onto a nitrocellulose membrane. Membranes were blocked for 1 h in 5% Topblock (Fluka) diluted in Tris-buffered saline supplemented with Tween 20 [150 mM NaCl, 10 mM Tris-HCl, 0.05% Tween 20 (v/v)] and incubated overnight at 4°C with anti PrP antibody POM1 (200 ng/ml). Horseradish peroxidase conjugated goat anti mouse IgG (H+L) (1:10.000, Invitrogen) was used as the secondary antibody and immunoreactivity was visualized using chemiluminescence (Luminata crescendo, Merck Millipore). (TIF) [file pone.0170503.s005.tif]

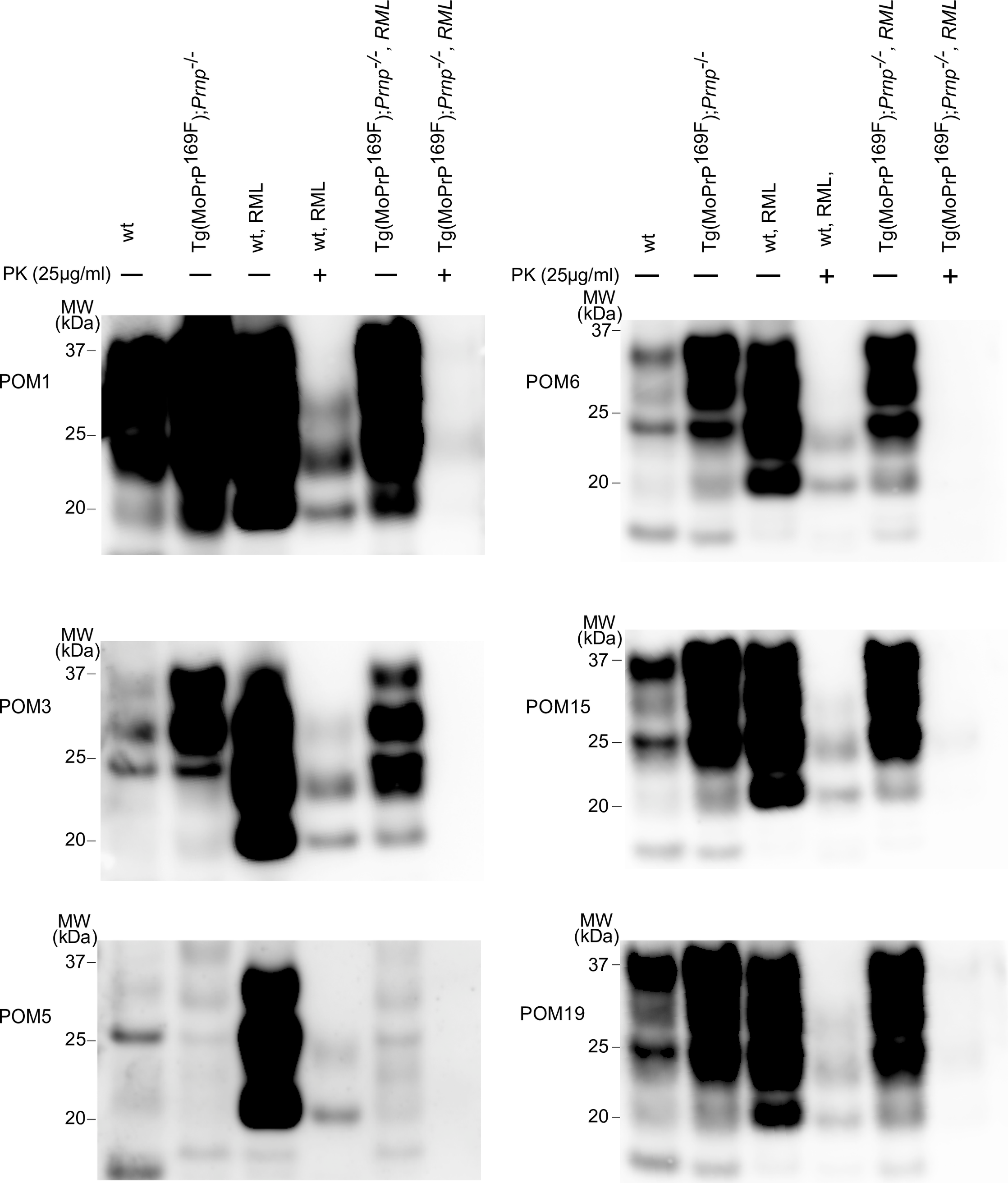

Supplement: S6 Fig — PK Western blot analysis of brain homogenates from RML-infected Tg(MoPrP169F);Prnp-/- mice probed with a panel of different anti-PrP antibodies (POMs), covering different epitopes of PrP, compared to brain homogenates from RML-infected wt Prnp+/+ mice. All antibodies detected increased sensitivity to PK digestion and no shorter PK-fragments for MoPrP169F prions. POM5 which recognizes the β2-α2 loop of PrP (residues 168–174) was not able to detect MoPrP169F, because of the point mutation in its recognition site. Brain homogenates from RML-infected wt Prnp+/+ mice were used as controls. 10 μg of total protein was treated with 25 μg/ml PK for 30 min at 37°C and loaded onto the gel. Non-digested samples from RML-infected wt and Tg(MoPrP169F);Prnp-/- were used as controls. Bands were detected with the different anti-PrP antibodies as indicated in the Figure at a concentration of 200 ng/ml. (TIF) [file pone.0170503.s006.tif]

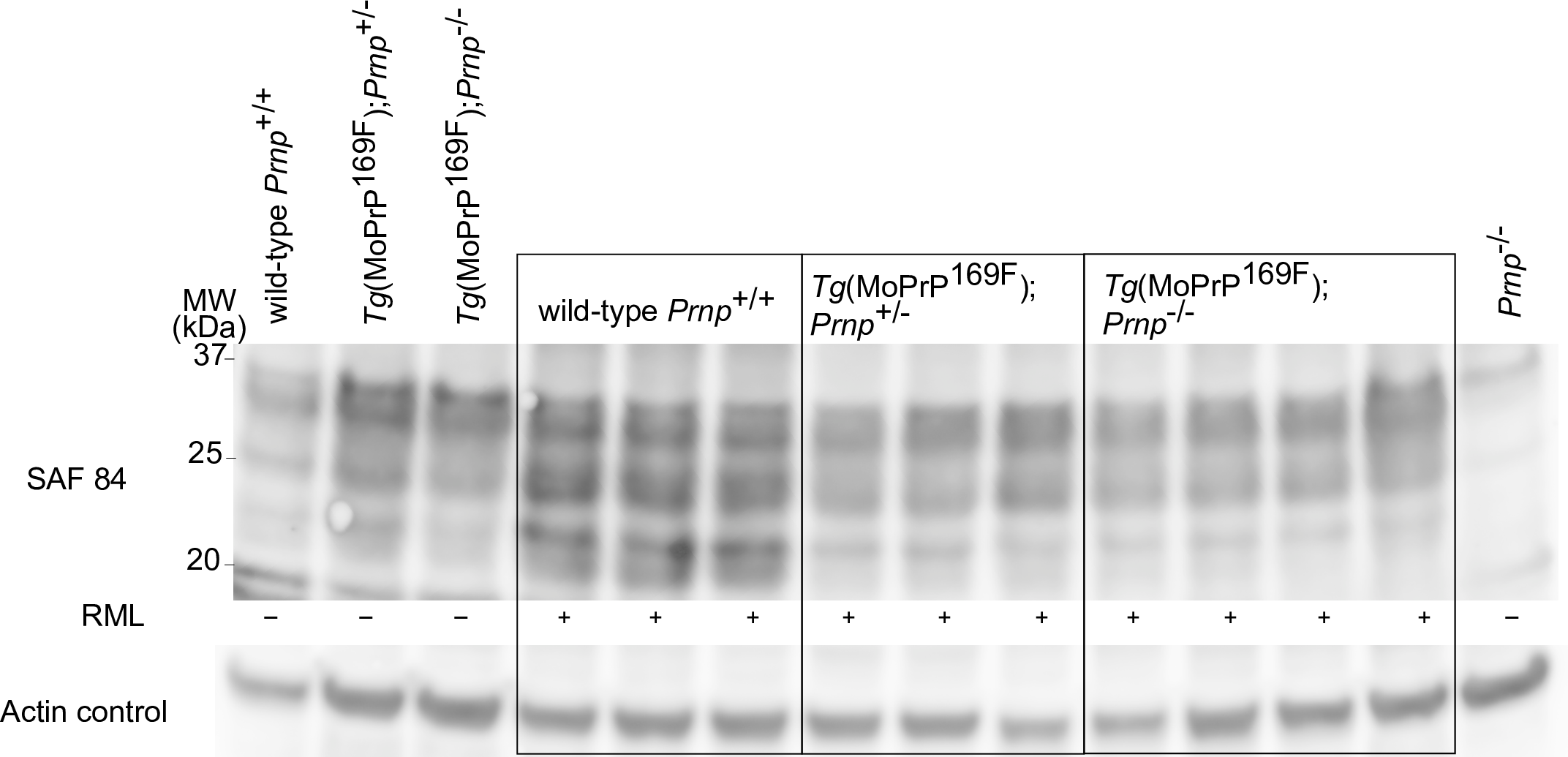

Supplement: S7 Fig — Western blot analysis of 10% brain homogenates (10 μg total protein per well) from wt Prnp+/+, Tg(MoPrP169F);Prnp+/- and Tg(MoPrP169F);Prnp-/- mice treated with or without RML. Bands in the PrP region differed from the Prnp-/- control. PrP was stained with the anti-PrP antibody SAF84 (1:1000). Actin is depicted as loading control (1:10000). (TIF) [file pone.0170503.s007.tif]

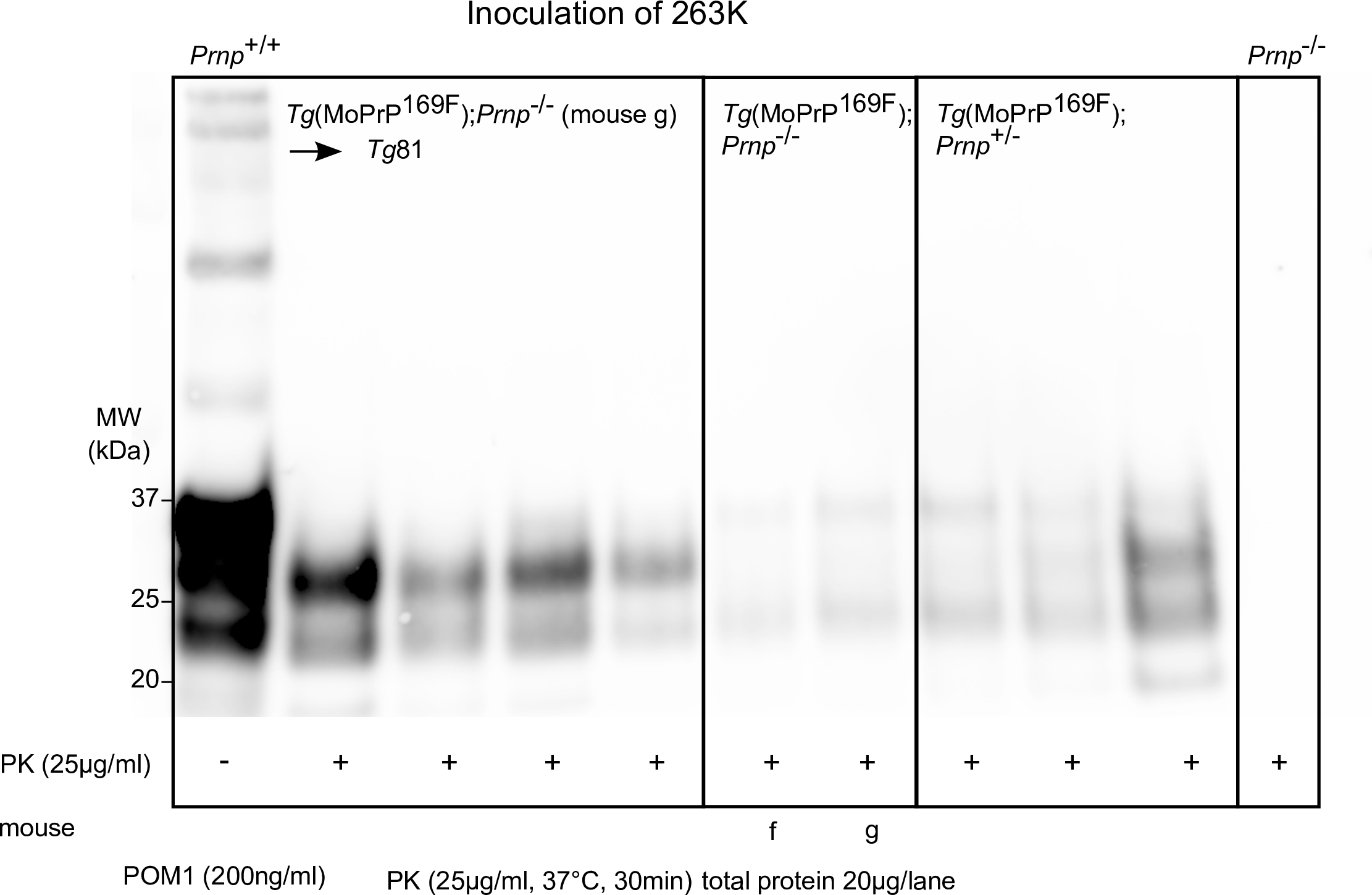

Supplement: S8 Fig — Passage of brain homogenate from 263K inoculated Tg((MoPrP169F);Prnp-/- mouse (g) (previously shown in Figs 2B and S3B) into hamster PrP expressing Tg81 mice led to death and accumulation of PK resistant PrP. In contrast Tg(MoPrP169F);Prnp-/- mice showed marked reduction of PK resistant material. In Tg(MoPrPF169F);Prnp+/- mice the amount of PK resistant material was also reduced compared to the passaged Tg81 mice, but showed some interindividual variability. 20 μg of total protein per lane was treated or not with 25 μg/ml PK for 30 min at 37°C. Bands were detected with the anti PrP antibody POM1 (200 ng/ml). (TIF) [file pone.0170503.s008.tif]

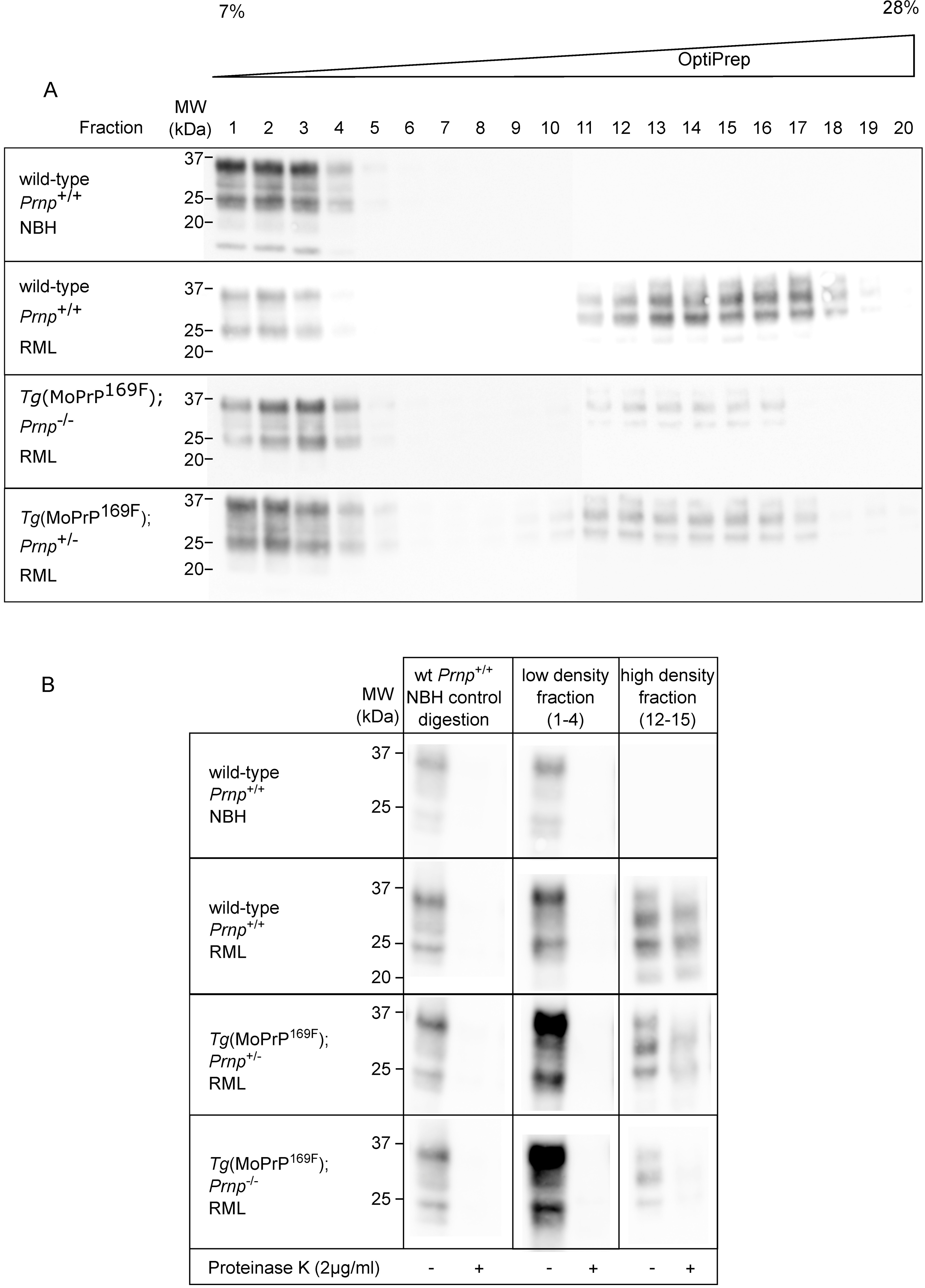

Supplement: S9 Fig — (A) Western blot analyses of total PrP from differentially fractionated brain homogenate samples of wt Prnp+/+ mice inoculated with noninfectious brain homogenate or RML and RML inoculated Tg(MoPrP169F);Prnp-/- and Tg(MoPrP169F);Prnp+/- mice to confirm data from Fig 5. A technical replicate of the data presented in Fig 5 is shown with all fractions (1–20) loaded to a SDS-PAGE from a 7–28% OptiPrep gradient. (B) Effect of proteinase K on PrP aggregates migrating in low and high density fractions. Western blot analysis of pooled low (fractions 1–4) and high (fractions 12–15) density fractions from the ultracentrifugation experiment show PK (2 μg/ml) sensitive PrP in the low-density fractions. In high density fractions, PK resistance is maintained in wt Prnp+/+, whereas reduced resistance is observed in Tg(MoPrP169F) mice. Fractions were methanol precipitated and adjusted to 190 ng of total protein per lane. PrP was detected using the anti-PrP antibody POM1 (200 ng/ml). (TIF) [file pone.0170503.s009.tif]

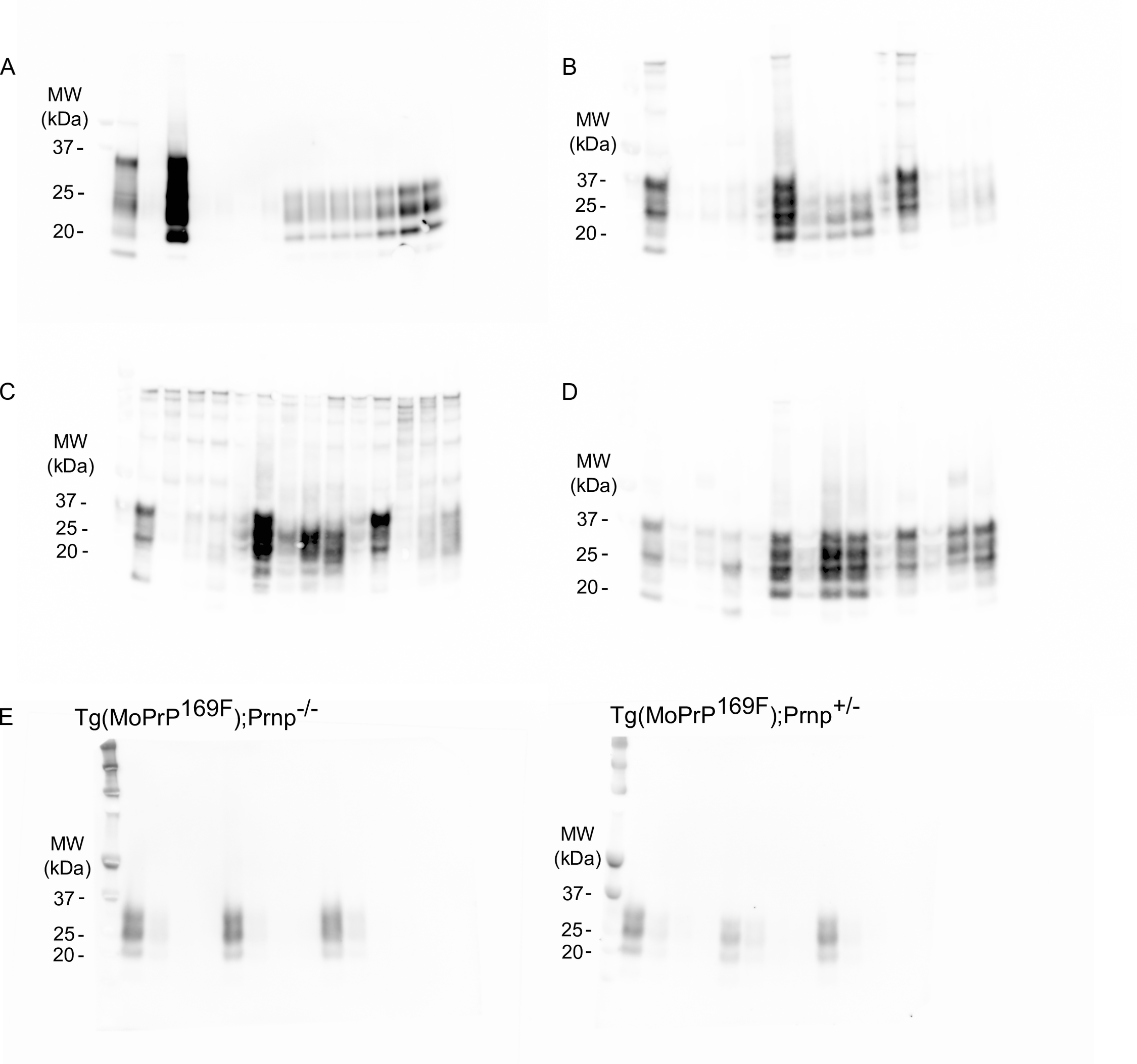

Supplement: S10 Fig — (TIF) [file pone.0170503.s010.tif]

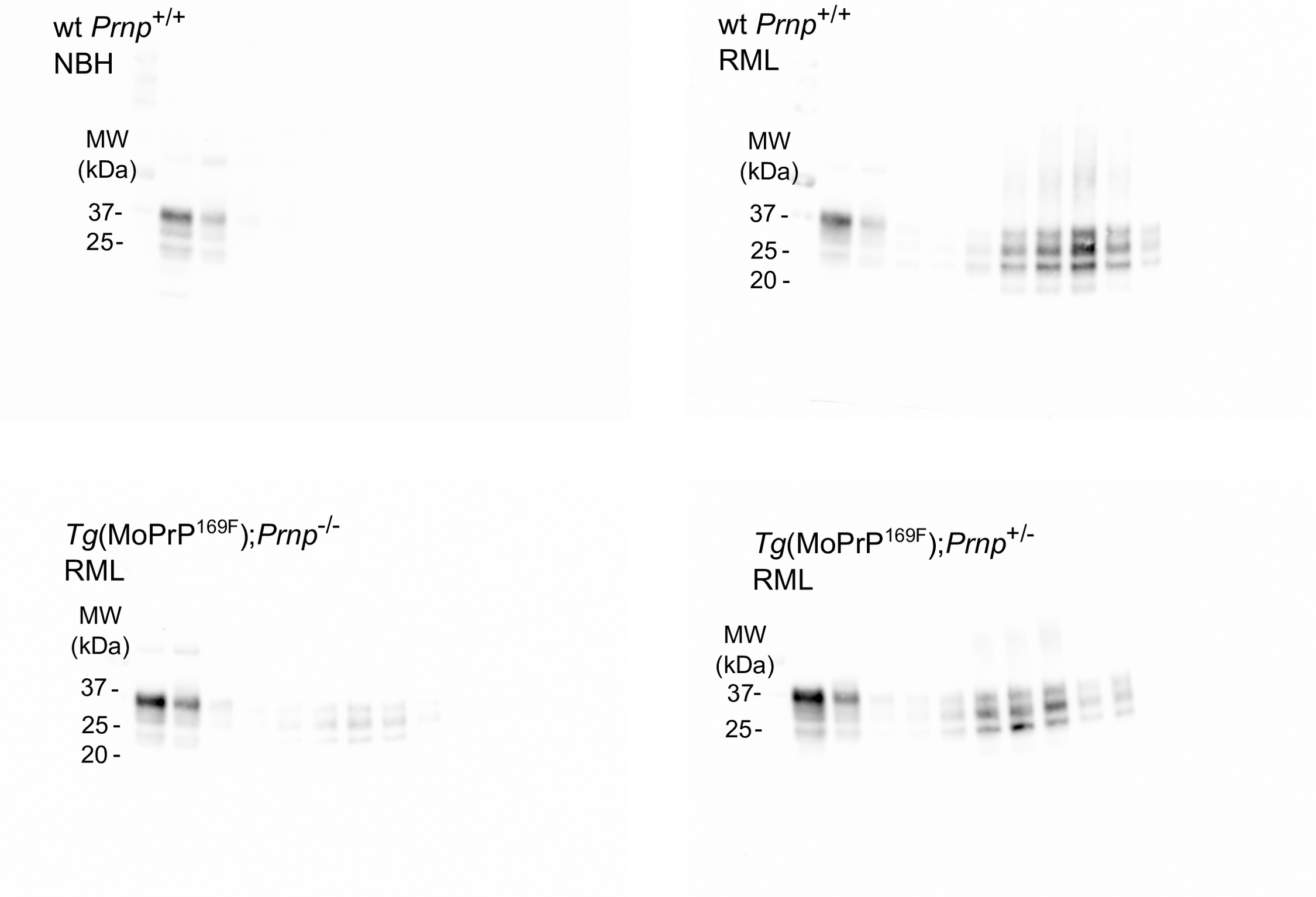

Supplement: S11 Fig — (TIF) [file pone.0170503.s011.tif]

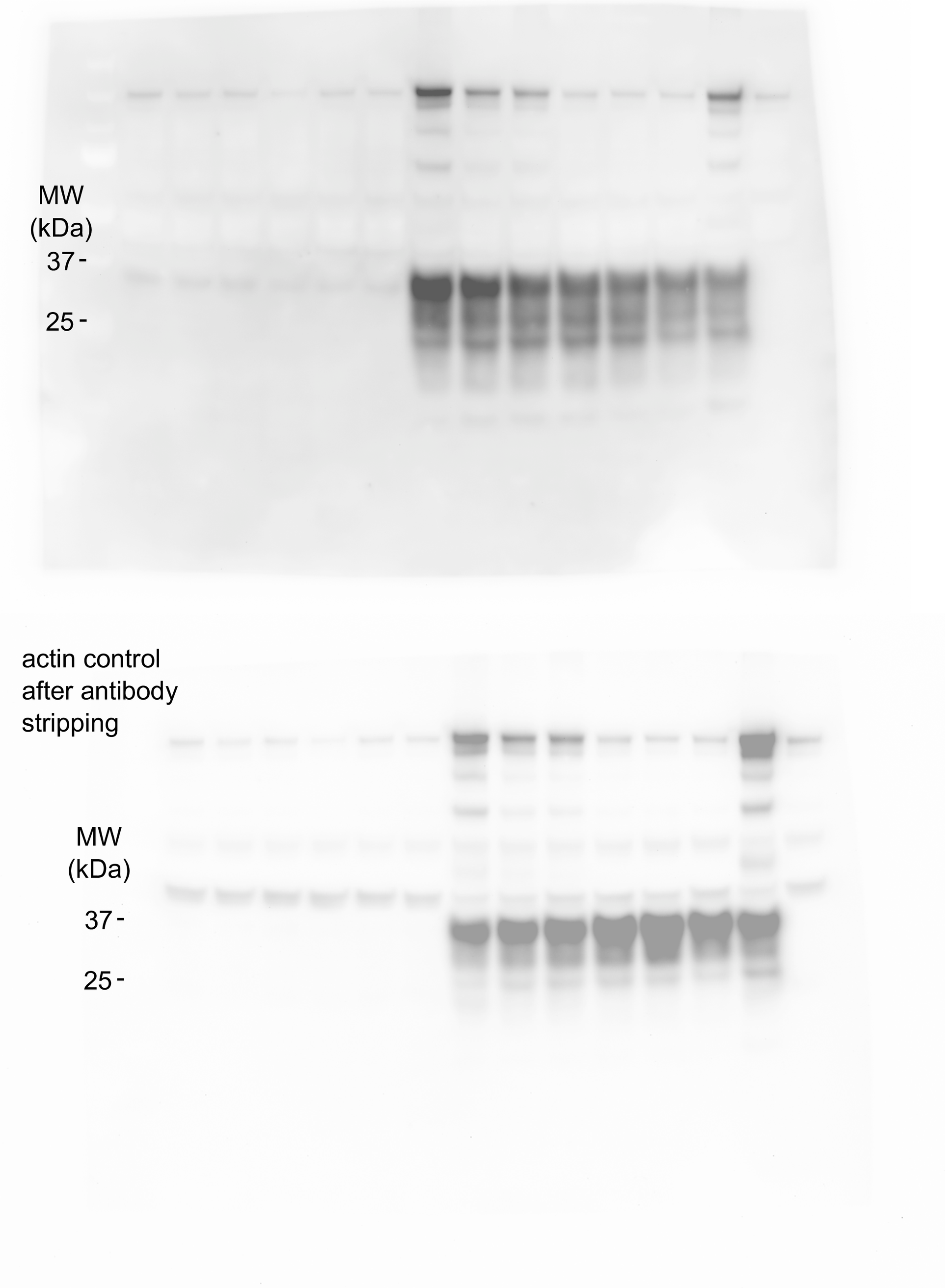

Supplement: S12 Fig — with size markers. (TIF) [file pone.0170503.s012.tif]

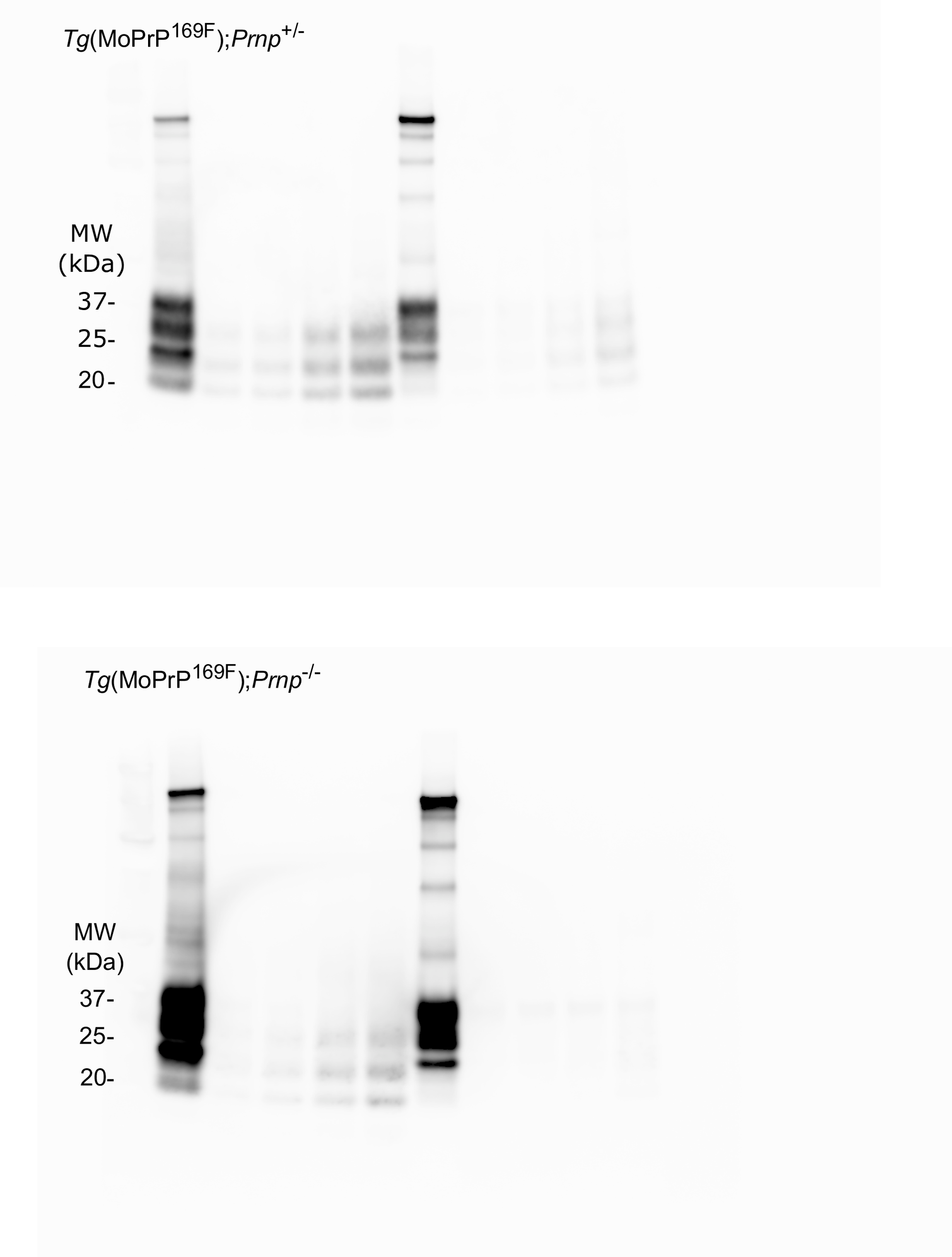

Supplement: S13 Fig — with size markers. (TIF) [file pone.0170503.s013.tif]

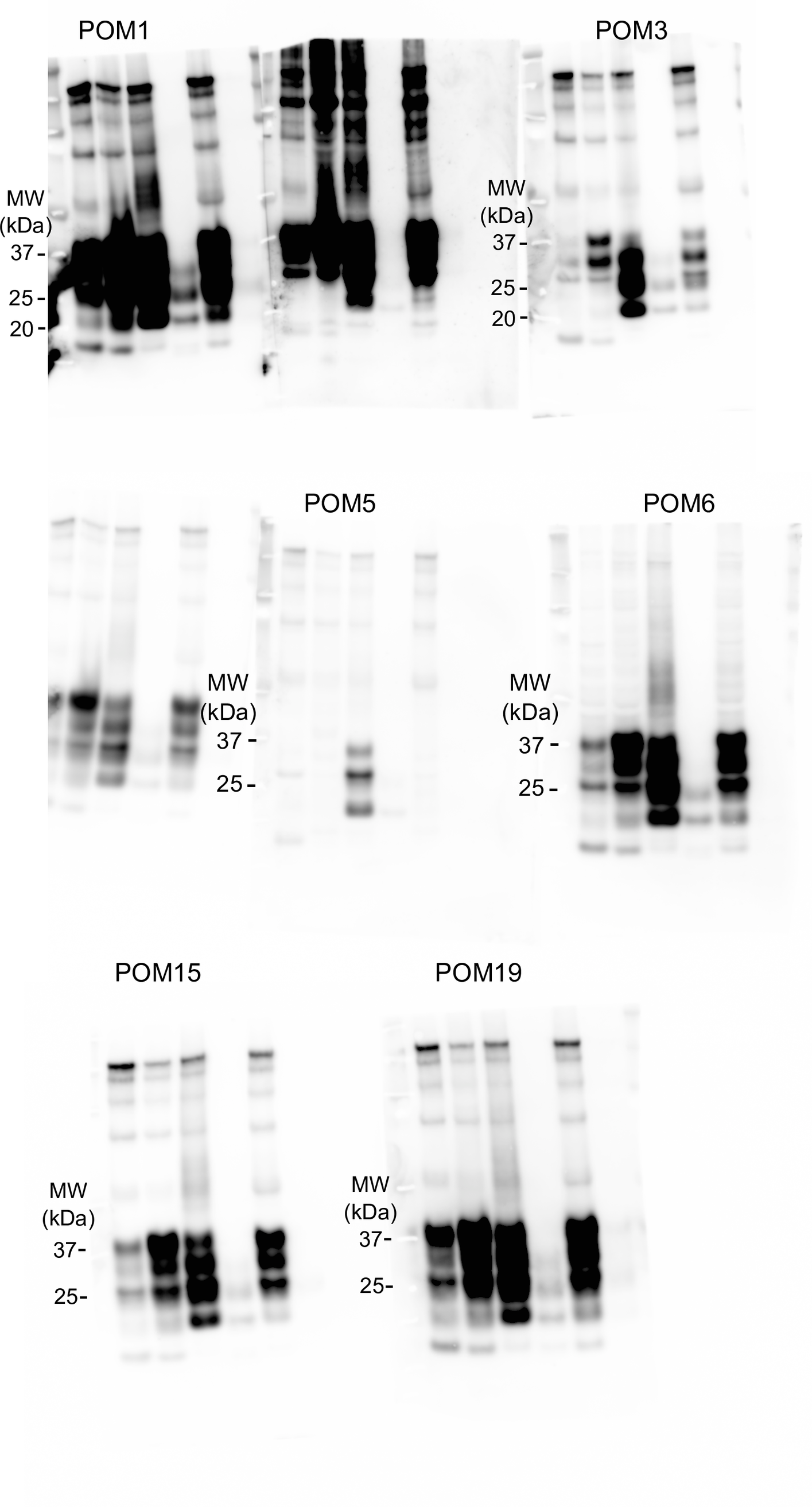

Supplement: S14 Fig — with size markers. (TIF) [file pone.0170503.s014.tif]

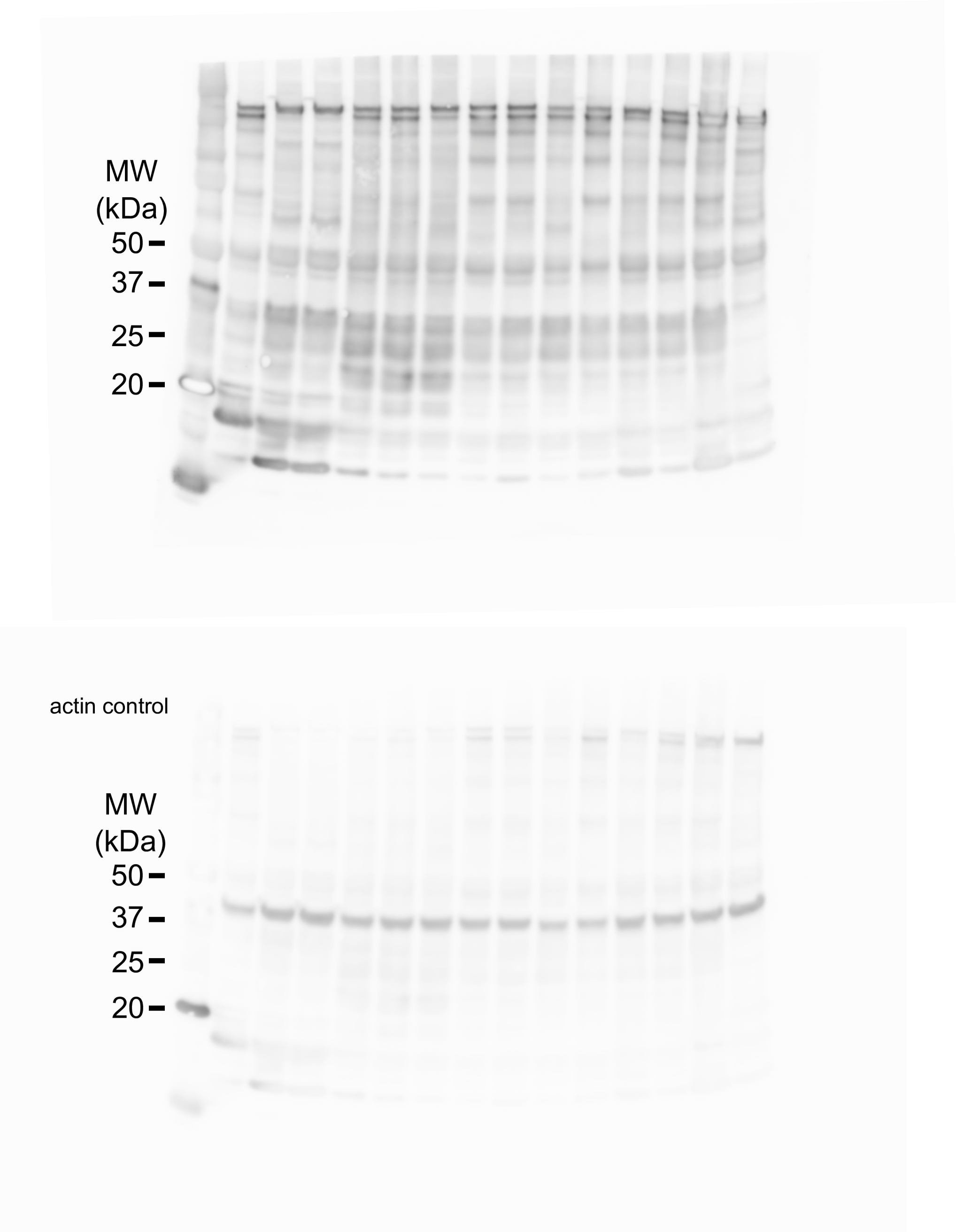

Supplement: S15 Fig — with size markers. (TIF) [file pone.0170503.s015.tif]

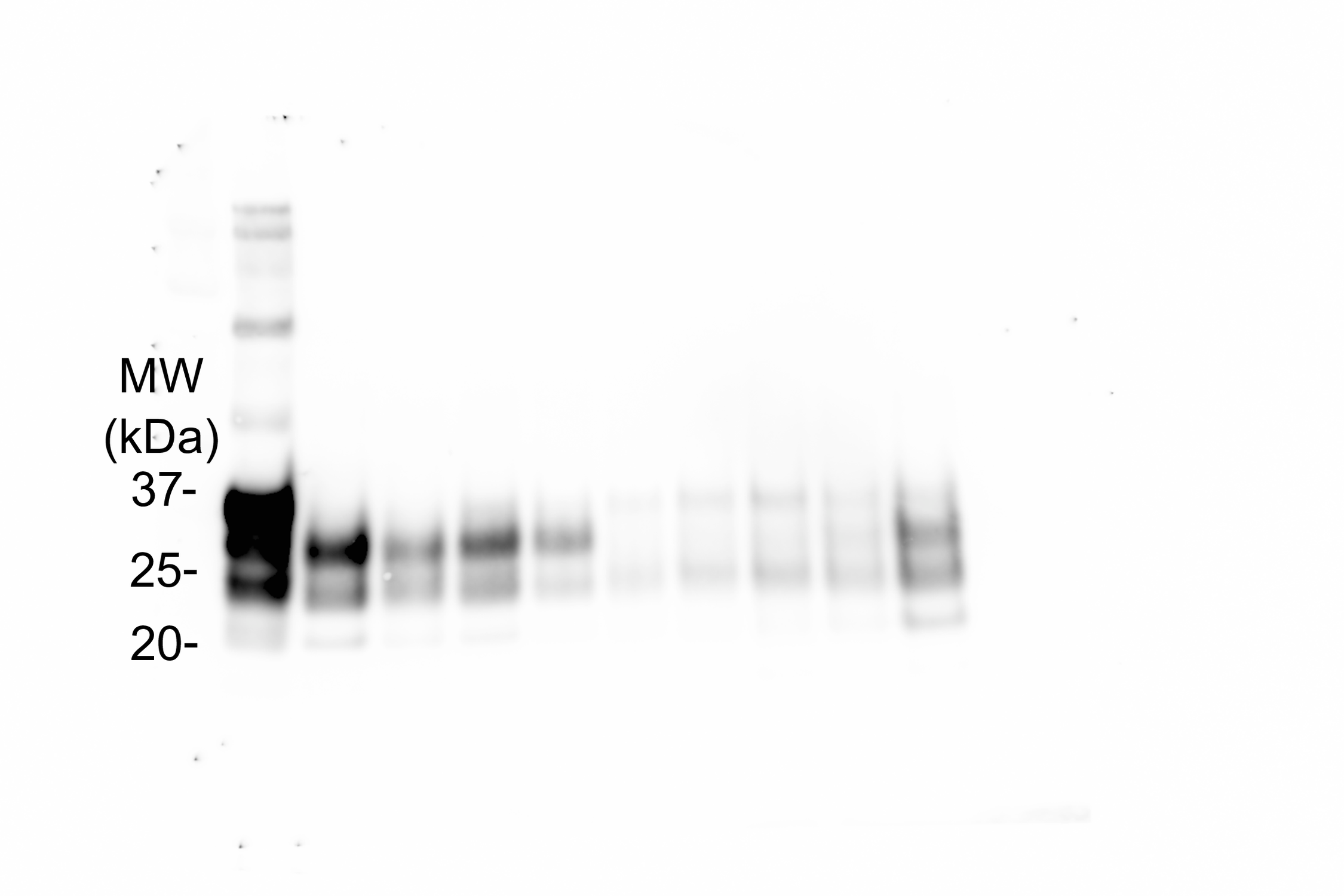

Supplement: S16 Fig — with size markers. (TIF) [file pone.0170503.s016.tif]

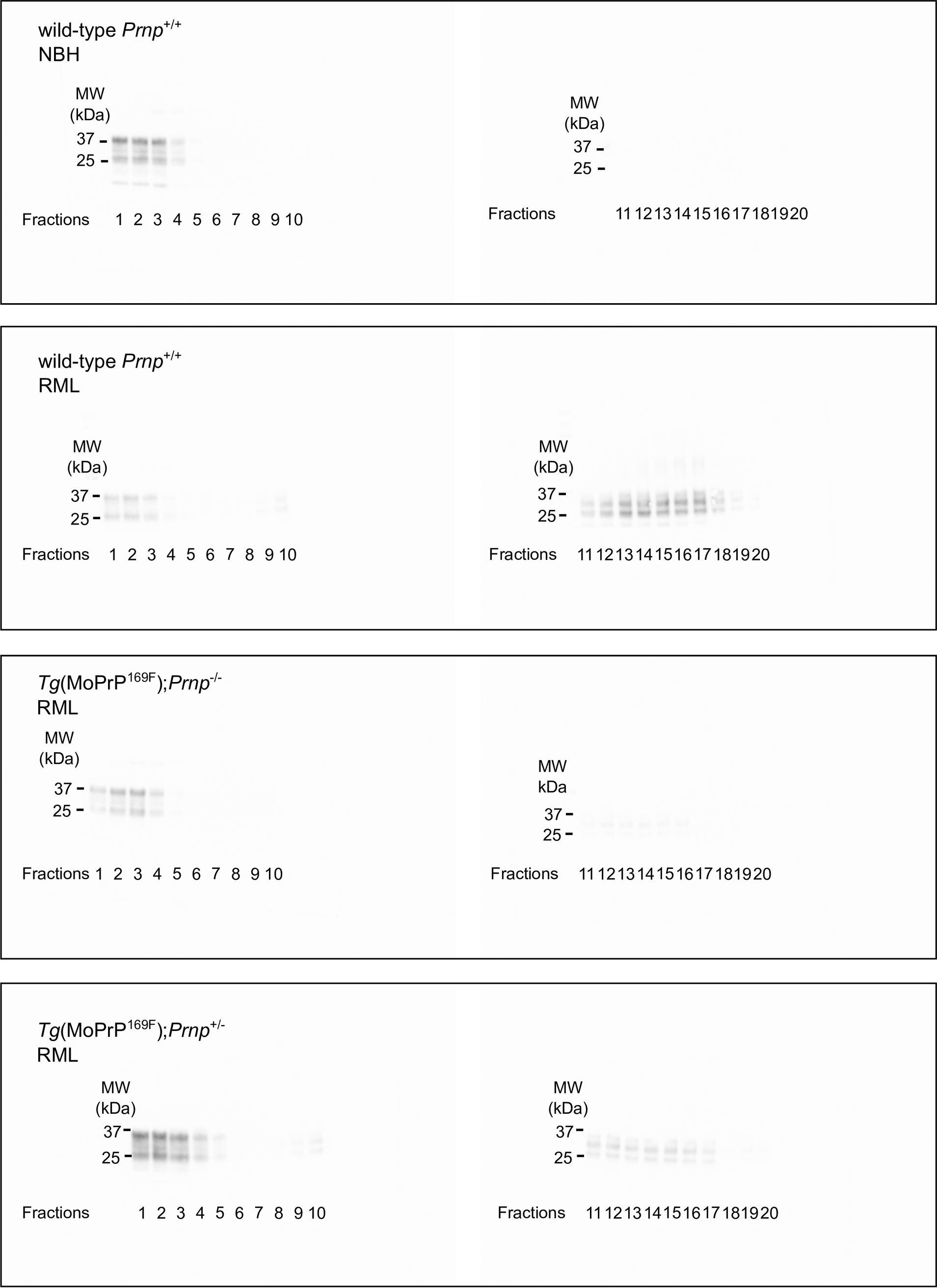

Supplement: S17 Fig — with size markers. (TIF) [file pone.0170503.s017.tif]

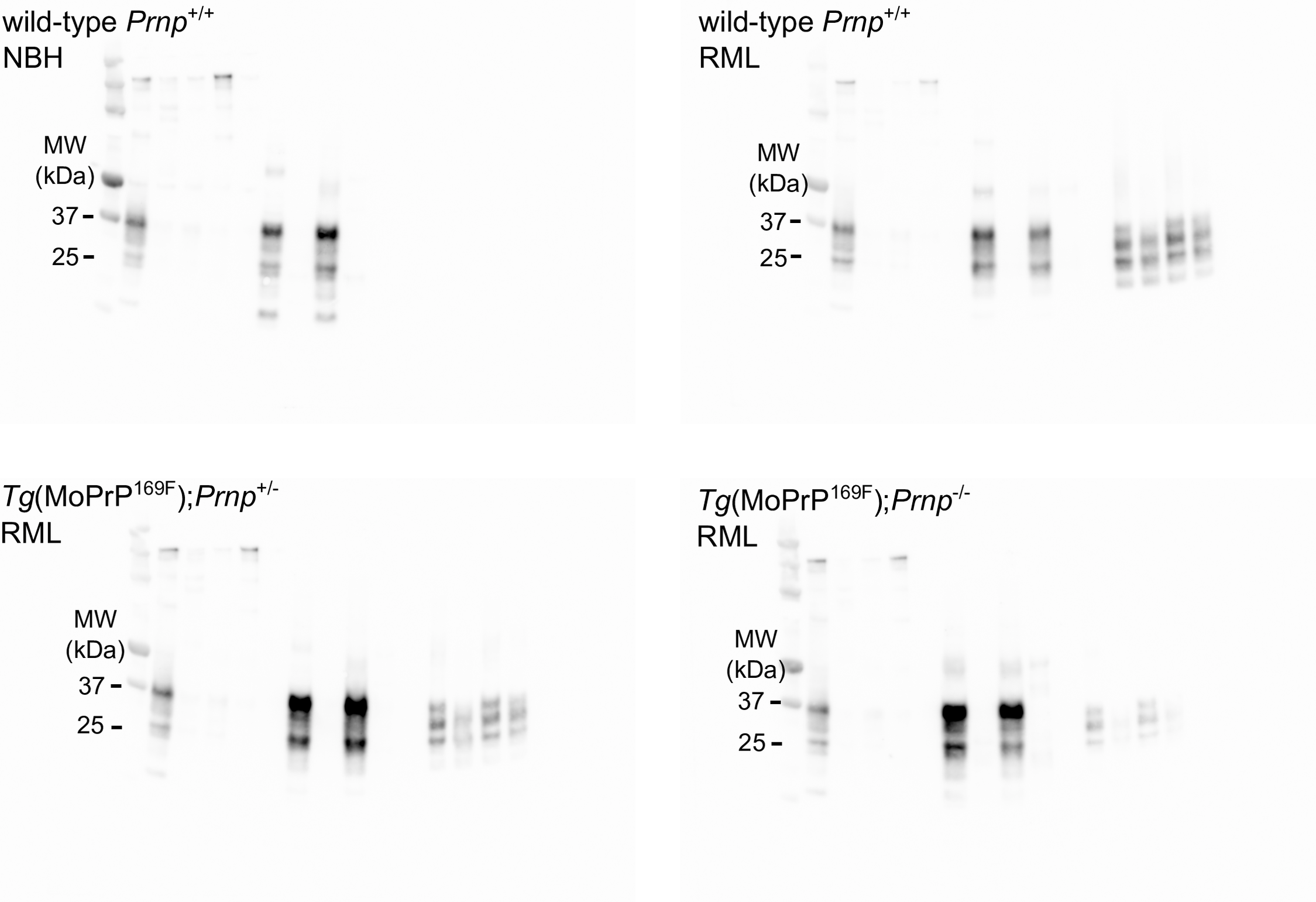

Supplement: S18 Fig — with size markers. (TIF) [file pone.0170503.s018.tif]
