# Supplementary material for: Protease resistance of infectious prions is suppressed by removal of a single atom in the cellular prion protein
Source: PLoS One. 2017 Feb 16;12(2):e0170503. doi: 10.1371/journal.pone.0170503 (PMC5313174; doi:10.1371/journal.pone.0170503)
Supplement: S1 Table — The mice were observed every other day after prion inoculation for clinical signs including gait, grooming, activity, rough hair coat, limb paresis and ataxia. Once the mice showed the first sign of scrapie (grade 1), they were monitored every day and wet food was supplied in the cage. When the mice reached score grade 2 that hampered the mice reaching the water bottle, they were euthanized by CO2 inhalation. (DOCX) [file pone.0170503.s020.docx]

**S1 table: Clinical assessment and scoring of mice inoculated with prions**

| **Score** | **Clinical signs** | **Assessment** | **Action** |
| --- | --- | --- | --- |
| 0 | No detectable signs of abnormal movement |  |  |
| 1 | Waddling gait, mild signs of reduced grooming, rough hair coat, limb weakness, front leg paresis* | Slight rolling while shaking the cage | Provide wet food in the cage;  Observe every day |
| 2 | Ataxia, reduced grooming and activity, paralysis, rolling* | Rolling while shaking the cage | Euthanize immediately if the clinical signs hamper the mice reaching the water bottle |
| 3 | Dead |  |  |

*: observed only in RML6-inoculated *Tg*a20 mice.
